# Tracing the evolution of the heterotrimeric G protein α subunit in Metazoa

**DOI:** 10.1186/s12862-018-1147-8

**Published:** 2018-04-11

**Authors:** A. D. Lokits, H. Indrischek, J. Meiler, H. E. Hamm, P. F. Stadler

**Affiliations:** 1 Neuroscience Program, Vanderbilt University, Nashville, TN USA; 2 Center for Structural Biology, Vanderbilt University, Nashville, TN USA; 3 Bioinformatics Group, Department of Computer Science, Leipzig University, Leipzig, Germany; 4 Computational EvoDevo Group, Bioinformatics Department, Leipzig University, Leipzig, Germany; 5 Chemistry Department, Vanderbilt University, Nashville, TN USA; 6 Pharmacology Department, Vanderbilt University Medical Center, Nashville, TN USA; 7 Center for non-coding RNA in Technology and Health, University of Copenhagen, Frederiksberg C, Denmark; 8 Institute for Theoretical Chemistry, University of Vienna, Wien, Austria; 9 IZBI-Interdisciplinary Center for Bioinformatics and LIFE-Leipzig Research Center for Civilization Diseases and Competence Center for Scalable Data Services and Solutions, University Leipzig, Leipzig, Germany; 10 Max Planck Institute for Mathematics in the Sciences, Leipzig, Germany; 11 Santa Fe Institute, Santa Fe, NM USA

**Keywords:** Heterotrimeric G protein, G protein coupled receptors, Evolution, Whole genome duplication, Paralog, Orthology, Genome annotation

## Abstract

**Background:**

Heterotrimeric G proteins are fundamental signaling proteins composed of three subunits, Gα and a Gβγ dimer. The role of Gα as a molecular switch is critical for transmitting and amplifying intracellular signaling cascades initiated by an activated G protein Coupled Receptor (GPCR). Despite their biochemical and therapeutic importance, the study of G protein evolution has been limited to the scope of a few model organisms. Furthermore, of the five primary Gα subfamilies, the underlying gene structure of only two families has been thoroughly investigated outside of Mammalia evolution. Therefore our understanding of Gα emergence and evolution across phylogeny remains incomplete.

**Results:**

We have computationally identified the presence and absence of every Gα gene (*GNA*-) across all major branches of Deuterostomia and evaluated the conservation of the underlying exon-intron structures across these phylogenetic groups. We provide evidence of mutually exclusive exon inclusion through alternative splicing in specific lineages. Variations of splice site conservation and isoforms were found for several paralogs which coincide with conserved, putative motifs of DNA-/RNA-binding proteins. In addition to our curated gene annotations, within Primates, we identified 15 retrotranspositions, many of which have undergone pseudogenization. Most importantly, we find numerous deviations from previous findings regarding the presence and absence of individual *GNA*- genes, nuanced differences in phyla-specific gene copy numbers, novel paralog duplications and subsequent intron gain and loss events.

**Conclusions:**

Our curated annotations allow us to draw more accurate inferences regarding the emergence of all Gα family members across Metazoa and to present a new, updated theory of Gα evolution. Leveraging this, our results are critical for gaining new insights into the co-evolution of the Gα subunit and its many protein binding partners, especially therapeutically relevant G protein – GPCR signaling pathways which radiated in Vertebrata evolution.

**Electronic supplementary material:**

The online version of this article (10.1186/s12862-018-1147-8) contains supplementary material, which is available to authorized users.

## Background

G protein Coupled Receptors (GPCRs) are a highly studied class of receptors due to their integral role in cellular signaling and therefore as therapeutic targets. Their evolution has shaped the chemical and biomolecular signaling systems of eukaryotes [1, 2]. Within this signaling cascade, a transducing element, the heterotrimeric G protein, composed of a monomeric α and obligate βγ dimer, acts as an intracellular relay for activated GPCRs to convert their message into an amplified signaling cascade. With only 16 paralogs in humans, compared to the 800 GPCR genes, the evolution of heterotrimeric G protein α subunit has received less attention than their transmembrane protein partners.

Shortly after their initial discovery and sequencing in several Mammalia species, the Gα subunit was found to be a highly conserved housekeeping protein [3]. As such, traces of genes encoding heterotrimeric G protein α subunits (*GNA*-) have been found in almost all major branches of Eukaryota [1, 4, 5] despite the proposed differences in GPCR and transmembrane receptor signaling mechanisms between the Unikonta and Bikonta lineages (see [1]).

Using only Mammalia sequences, the first theory of G protein α evolution posited the relative evolution of four of the five Gα families (Gαi, Gαq, Gαs and Gα12; Gαv having not yet been discovered) [3]. Focusing on the development and radiation of the visual system, others have evaluated the evolution of transducins (*GNAT1* and *GNAT2*) and other critical protein-coding genes in the vision signal transduction pathway in both rods and cones across Vertebrata and non-vertebrate Chordata [6–9]. However, to our knowledge, there have been no reports focused on studying the evolution of the other three families of Gα in Deuterostomia with the exception of Gα subunits in the fish chemosensory systems [10], and a more recent, coarse-grained study evaluating paralog counts across Opisthokonta phylogeny [5].

From these studies and others, we have compared our estimation of when each paralog emerged within Metazoa evolution. We have found numerous differences in the timing and number of predicted gene gain and loss events, due to a) differences in methodologies employed while searching for paralogous sequences and constructing phylogenetic trees and b) increased search space through the inclusion of more genomes. In addition to reporting new and manually curated gene annotations, we have also uncovered variations in alternative splicing patterns, non-canonical splice sites (SS), novel intron gain and loss events, Primates gene retrotranspositions and subsequent pseudogenization, as well as other nuanced deviations to the gene structure of this family. These data allow us to present an updated view on G protein α subunit evolution.

## Methods

### ExonMatchSolver

Genomes were analyzed for curated annotation within the ExonMatchSolver (EMS) framework according to its *Implementation and Usage* [11] utilizing both paralog-specific, individual translated coding exons (TCE) and full paralog sequences. Briefly, the EMS pipeline utilizes TCEs as the fundamental building blocks for its searches. Paralog-specific TCE amino acid (AA) sequences of a close relative to the target species were utilized as the query against the target genome. There are 16 *GNA*- genes within humans. As each family was expected to have a conserved exon-intron structure throughout Metazoa, the high quality annotations of human *GNA*- genes were utilized as the initial templates. Sister groups of Mammalia were evaluated next, before moving on to more distant families. For each major clade (Sauropsida, Amphibia, Actinopterygii, etc.), curation began within the species assembly with the highest reported sequence coverage, genome quality and level of annotation. This curated sequence was used as a seed TCE query for further analysis within that clade. A minimum of two orthologs were used as individual inputs for the *hmmsearch* when querying each target assembly. In addition to exon border position information, EMS also utilizes full-length protein sequences to annotate orthologous proteins along the target genome assembly via a spliced alignment [11]. A minimum of two orthologs from closely related species were utilized as protein sequence queries for the target spliced alignment.

### Data sources

**Fig. 1:**
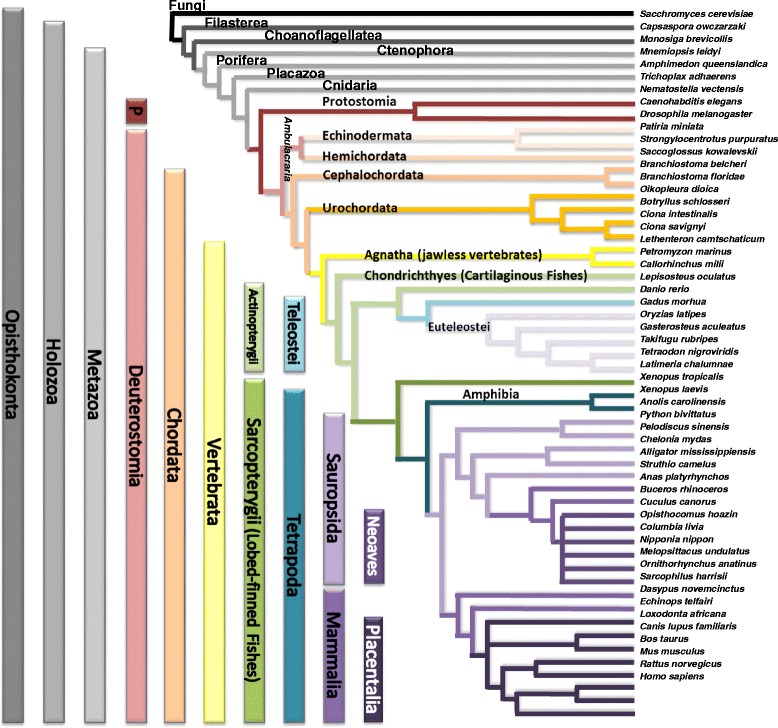
All phylogenetic branches investigated. 45 species of Deuterostomia were evaluated through the EMS pipeline. The Latin names and clades for each species are provided. The outgroups include two Protostomia species, four non-Bilatera Metazoa, two Non-Metazoa Holozoa and one Fungi. Protostomia and Deuterostomia together form the group of Bilateria. Echinodermata and Hemichordata form the group of Ambulacraria

A total of 65 species were evaluated; 45 of which were directly assessed through the EMS pipeline for curated gene annotation (see Additional file 1: Table S1); the additional species were utilized for supplemental assays as described. All queried genomes were obtained from public repositories [12–18]. The latest version of each genome was utilized for all analyses unless otherwise noted (*as of October 2016*). All major phylogenetic clades of Deuterostomia were investigated with the EMS pipeline, when genomes were available (Fig. 1). We included representatives of the following clades as outgroups to our analyses: Protostomia (2), non-Bilateria Metazoa (4), non-Metazoa Holozoa (2), non-Holozoa Opisthokonta (1). To reflect the orthology relationship, all *GNA*- genes which predate the radiation of Vertebrata are denoted as *preGNA*- for clarity, as recommended by the HUGO convention of gene names [19].

We utilized the Ensembl genome browser [15, 18, 20] and NCBI’s genome and assembly browser [16] for our starting queries as these databases contain easily accessible and high quality genome annotations. To validate gene gain and loss events, we evaluated the transcriptome shotgun assembly (TSA) sequence database, expression sequence tag (EST) database, and UniGene databases, accessed through NCBI [16, 17, 21, 22], using amino acid-based (*tblastn*) search queries. It is important to note that tissue-specific expression of some paralogs may hinder sequence validation through this approach. Synteny information (co-localization with neighboring genes) was also utilized in evaluating paralog assignments and gene loss, when available, through the Ensembl and NCBI genome browsers. The species tree that was used for mapping gene gain and loss events (Fig. 1) is based on screening of recent literature and the consensus therein [23–26].

### Reconstruction of gene trees

In order to build phylogenetic maximum likelihood (ML) trees on the nucleotide and amino acid level using RAxML protocols [27, 28], exonic, protein-coding sequences of interest were aligned using both ClustalOmega [29] and MUSCLE [28], and edited with the Jalview alignment editor [30]. The Jalview alignment editor was utilized to manually inspect the MSAs to ensure annotated exon border positions were maintained during ClustalOmega and MUSCLE alignments. Additional files of the edits before and after Jalview inspection have been provided as Supplemental files X and Y. MSAs were then handed over to RAxML [31]. The appropriate amino acid or nucleotide substitution model for each tree was determined through Prottest [32] and additional tree parameter optimizations were conducted through preliminary rounds of ML searches comparing the different models of rate heterogeneity available in RAxML, respectively (Gamma, CAT, and a variable heuristics optimization [27, 33]). Random starting trees were also employed for initial independent ML tree searches to determine if random starting trees improved topology search space over a maximum parsimony starting tree. After optimizing the substitution model with the best model of among-site versus per-site heterogeneity rates and starting tree, the ML trees were compared for their diversity across tree topology. The strength of the phylogenetic signal was assessed through comparison of the best likelihoods, and pairwise-Robinson Fould (RF) distance calculations were conducted across all independent searches. Production runs calculated support values for all ML trees and utilized *bootstopping* for all bootstrap replicates to decrease computational time. Bootstrapped replicates were summarized into Extended Majority Rule Consensus Trees and reported with bootstrap (BS) values as additional files (Additional file 2: Supplemental file 1, Additional file 3: Supplemental file 4, Additional file 4: Supplemental file 5 and Additional file 5: Supplemental file 6). Pairwise-RF distance calculations across topologies as well as a Shimodaira and Hasegawa test were used to confirm that differences between likelihoods were not significant before summarizing into consensus trees.

### Gene tree-species tree reconciliation

NOTUNG v.2.8.1.7 [34] was utilized to reconcile the known species tree as extracted from timetree [35] with the bootstrapped maximum likelihood gene tree generated by RAxML including all Holozoa species investigated. The root was chosen randomly from a set of roots proposed by NOTUNG which minimizes the gain/loss event score. After rearrangements, NOTUNG reconciled the species tree with 100 duplications and 209 losses (Edge Weight Threshold: 90.0). The number of duplications and losses can be over predicted in cases when the gene tree topology does not correspond to the species tree topology. In our study, the fast divergence of a paralog in different clades and missing sequence data may also contribute. We further considered more information (synteny, timing of WGDs) that was not available to NOTUNG. Those proposed, additional duplications are not discussed in detail within the main document, but may be inspected in detail.

### Investigation of protein-binding motifs within DNA/RNA sequences

Centrimo [36] was used to perform a local (positional) enrichment analysis of in vivo and in vitro DNA- and RNA-binding protein (DPB/RBP) motifs from the following databases: Ray 2013 restricted to available Vertebrata motifs (human, mouse, frog) [37], Jolma 2013 [38], Jaspar Core database 2014 [39], BS Uniprot [40] mouse. Centrimo evaluates absolute enrichment of a motif by performing a binomial test to determine whether the best match motif counts at a specific position are significantly different from a uniform motif distribution. Centrimo was also run in differential mode to conduct a Fisher’s exact test to determine positional motif enrichment in a primary sequence set in comparison to a control set (adjusted *p*-value corrected for multiple testing < 0.05 for both tests).

First, the potential overlap of all conserved non-canonical splice sites (SS) (the 5′ ‘GC’ SS of intron6 in *GNAI1*, and the 3′ ‘TG’ SS of intron3 in *GNAS*) with DBP/RBP motifs were interrogated by testing differential motif enrichment in the nucleotide sequence surrounding the SS (full-length exon sequence and 40 nt of the intronic sequence). All orthologous sequences in the query set conserved the non-canonical SS, while the control set contained sequences with the canonical SS at the orthologous position. Second, the positional enrichment of potential DBP/RBP motifs was investigated within exon3 of *GNAS* and the surrounding conserved region by performing an absolute, local enrichment test. Homologous sequences were extracted from an additional 27 Placentalia from the Ensembl webserver [15] to form a total dataset of 33 species.

### Detection of Retrogenes in Primates

The longest protein-coding isoform of each human *GNA*- gene was blasted against the human genome. Sequence matches overlapping annotated retrogenes were extracted at the nucleotide level via the Ensembl webserver [15] (*GNAI2P2*, *GNAI2P1*, *GNAQP1*, *GS1-124 K5.9*, *RP11-611O2.6, AC010975.2*, *RP11-100 N3.2*). 11 target Primate genomes (Additional file 6: Figure S1) were then queried using these human *GNA*- pseudogene annotations. Primate retrogenes were retrieved as single blast hits with the following settings: *blastn*; e-value < 10^− 5^; match/mismatch: 1, − 3; and opening/extension: 5, 2. Additional synteny (gene co-localization) information was also considered when identifying potential retrogenes. In cases with short scaffold lengths and no available synteny information, full-length parent genes were re-blasted against the putative target loci. Loci that retrieved multiple, subsequent sequence matches were then excluded. A single sequence match was considered to be an individual exon of a multi-exon paralog if it covered less than 50% of the query sequence. Cases of 30–50% query coverage were manually inspected to identify exon borders.

Conserved open reading frames (ORFs) between orthologous retrogenes that showed similarity to the multi-exon paralog were interrogated. These potential ORFs within the retrogene loci (Blast hit +/− 300 nt) were identified with ORF Finder [41] and similarity to the parent protein confirmed by blast (bl2seq –n blastp). Then potential novel ORFs with coding potential that were not similar to the parent protein sequence were investigated. For this purpose, the retrogene loci were aligned with ClustalOmega [29] and coding potential was accessed with RNAcode [42] probing at least four different reference species. Sequence hits were reported if the region was conserved in all Primates and contained at least one methionine as a possible initiation codon for translation.

Expression of pseudogenes was investigated utilizing the following recourses: the Ensembl genome browser [15, 18], the USC genome browser (with available species-specific mRNA, EST, cDNA and protein data) [43], the Expression Atlas (release 18 06 2017) [44], and psiCube [45]. In order to search the Expression Atlas, we only considered those 16 pseudogenes of non-human Primates that had Ensembl gene IDs of the orthologous pseudogene (RPKM > 0.5). Only a selection of the datasets, which showed expression of the pseudogenes are presented.

### Detection of natural selection in *GNAO*

The branch-site model implemented in CODEML in the PAML package [46] was utilized for the identification of residues within branches under positive selection. Significance was tested by comparing to the χ^2^ distribution. To exclude possible biases from codon model choice or shifts in GC content, three different codon models were applied (Codon Table, F3X4 and F1X4) and were assessed for consistency between results. Residues under positive selection were identified by Bayes Empirical Bayes (BEB) analysis [47]. The respective alignments were tested for the presence of recombination with the RDP4 software [48] in order to minimize false positive signals of positive selection that are caused by other processes (linear sequence = TRUE, Disentangle overlapping events = TRUE). All recombination tests results were not significant (default values used, *p* < 0.05). To obtain estimates of the robustness of model parameters, we performed 100× bootstrapping with the codeml_sba software for those branch-site tests that rejected neutral selection in class 2a and 2b in the foreground branch (*p* < 0.05) [49, 50].

A phylogenetic tree was constructed for the concatenation of exons7 and 8 of all *GNAOs* including Cephalochordata and Vertebrata (excluding Teleostei and Agnatha) and evaluated with two different foreground branches: the ancestral branch of *GNAO.1* and *GNAO.2* after the exon duplication, but preceding speciation of Vertebrata, respectively (see Fig. 9). The respective nucleotide sequences were aligned with MASCE v1.01b [51]. Sequences with missing data in these exons were excluded. The divergence of this alignment is not ideal (tree length 15.7 in H0, F3X4). However, as high divergence would lead to a loss of power rather than an increase in the rate of false positives in the test [52], the divergence is not considered to be deleterious to the analysis. Positive selection and differences in selection pressure were also tested in the foreground branch of a gene tree composed of *GNAO* (*a*,*b*)*.1* s and *GNAOa.2* sequences including exons7 and 8 of Actinopterygii (ray-finned fishes). Foreground branches were defined as the branches after the 3R WGD and before Teleostei speciation (ancestral branches of *GNAOa.1*, *b.1* and *a.2*, respectively, see Fig. 9).

### Computational modeling of tertiary structures

Available crystal structures of Gα subunits and structural models based on crystal structures were utilized to map exon sequence positions onto tertiary folds. Though all structures and models utilize Mammalia sequences, the highly conserved tertiary and exon-intron structure of Gα supports that the relative exon position mappings are maintained across all phyla. The crystal structures of Gαq bound to PLCβ3 and RGS8 were utilized (PDB ID 4QJ3 [53] and 5DO9 [54], respectively). The active monomer of Gαs (PDB ID 1AZT [55]) was used in addition to the crystal structure of Gαi bound to Gβγ (PDB ID 1GP2 [56]) and to RGS4 (PDB ID 1AGR [57]). Comparative models of Gαo (human *GNAO.1* transcript variant) and Gαs (human sequence without exon3 and extended exon4) were constructed from previous modeling studies of the ternary complex [58] (activated GPCR bound to Gαi and Gβγ) by replacing Gαi side chain residues with either Gαo or Gαs sequence while maintaining backbone atom coordinates. After threading these sequences, model hybridization continued with optimizing fragment insertions, and relieving chain breaks through the comparative modeling RosettaCM protocol [59]. The relaxed and optimized structural models were then utilized for further exon sequence mapping based on conserved sequence positions. All crystal structures and models were visualized with Pymol [60].

## Results and Discussions

### Gα paralog evolution before the 2R WGD of Vertebrata

#### *preGNA-* genes before the 2R WGD

The early Vertebrata ancestor underwent multiple rounds of whole genome duplication (WGD) [61–64]. These events allowed for increased gene number and sequence diversity and are thus of special interest. Therefore, we primarily focused our study to species of Deuterostomia, but included nine non-Deuterostomia Opisthokonta as outgroups. To clarify the orthology relationship the following gene names are used to refer to the progenitor representatives of the Gα families before the Vertebrata radiation: *preGNAI*, *preGNAO*, *preGNAQ*, *preGNAS*, *preGNAV*, *preGNA12* with the exception of paralogs within *S. cerevisiae* which are referred to as *GPA1* and *GPA2*.

Using the EMS gene annotation pipeline, we report an updated, full account of paralog presence and paralog assignment within the outgroup species in comparison to previous reports. We find seven *preGNA*- paralogs in *C. owczarwaki* (previous reports find eight [2, 5]), six in *A. queenslandica* (previous studies report a range from five to seven [2, 5], while we and [2] identified eleven paralogs in *M. leidyi*. [5] report twelve to thirteen). All reports within *M. brevicollis* and *S. cerevisiae* were found to contain three *preGNA*- and two *GPA*- paralogs, respectively.

We identified gene sequences for all five primary families (i, q, v, s and 12) in Ctenophora, Porifera, and Cnidaria (non-Bilateria Metazoa); four families were confirmed in Placozoa (i, q, s, and 12) and Filasterea (i, q, v, and s) while only two families were present in Choanoflagellatea (v and 12) (Table 1, Additional file 7: Supplemental file 2). We conclude that the five known primary families of the Gα subunit existed before the emergence of Metazoa in the Holozoa ancestor, though species-specific deletions exist (see Appendix A.i for lineage-specific tandem duplication events).

**Table 1:**
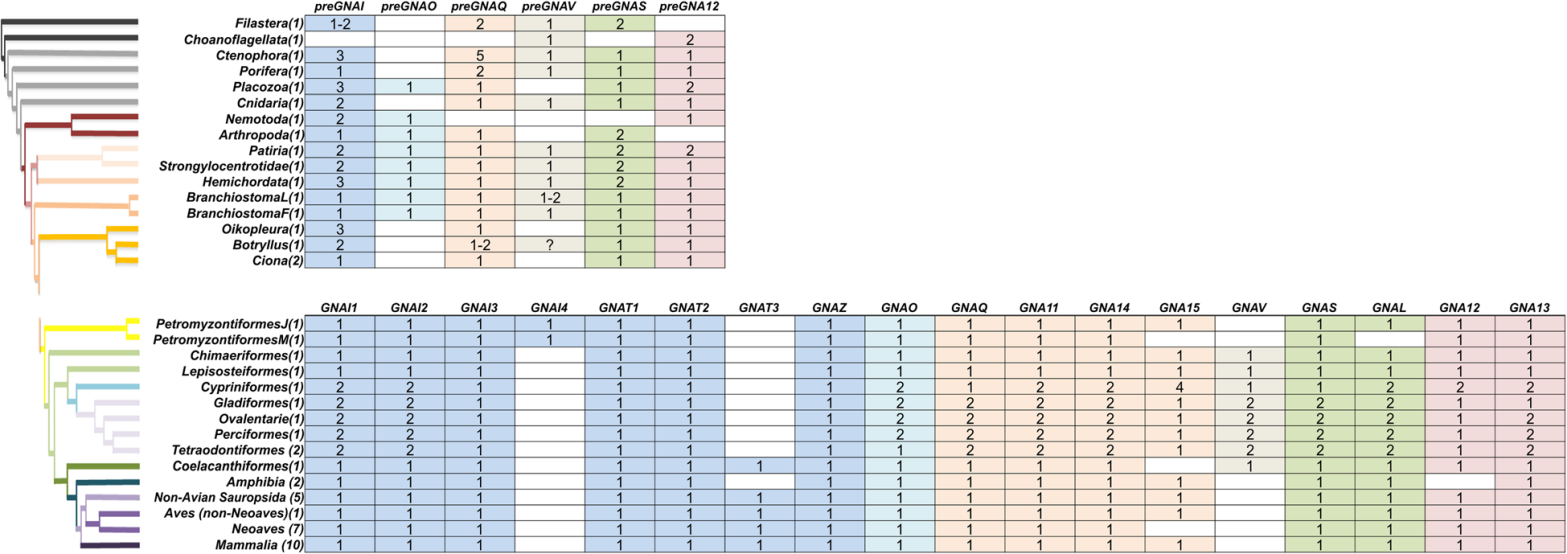
(pre)*GNA*- paralog presence before and after the 2R WGD in Vertebrata projected onto a Deuterostomia species tree

**Fig. 2:**
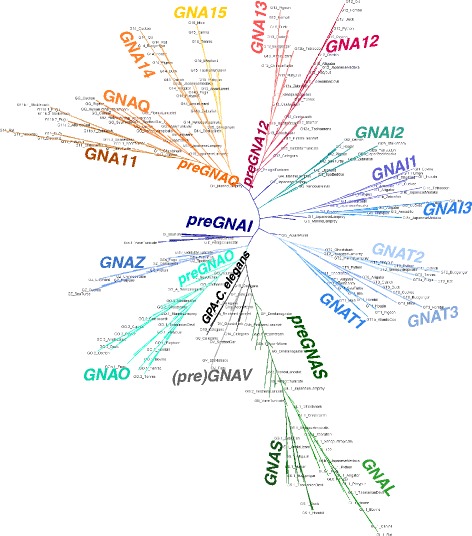
Maximum Likelihood Tree of (*pre*)*GNA*- genes. ML tree built with all paralogs and sequences evaluated. The tree is also included as separate file with BS values in Nexml format Additional file 2: Supplemental file 1. See Additional file 1: Table S1 for taxonomic groups. A reconciled, rooted gene tree of all evaluated (*pre*)*GNA-* genes in Holozoa is included as with reported bootstrap values of > 90% with all *preGNA-* genes denoted in black. The inference of gene duplications based on the gene tree and species reconciliation are in accordance with the hypotheses discussed herein. Major differences are indicated otherwise

We clarified the identity of previously unclassified and/or ambiguous sequences of non-Deuterostomia Metazoa, allowing an improved depiction of Gα emergence [5]. In addition, we also found evidence to support *preGNAO*-like sequences in Protostomia and Placozoa, but not within Cnidaria, contrary to a previous report [5, 65]. We do not find evidence of the three previously reported *preGNAO* paralogs outside of Metazoa [5]. The Gαi family was thus represented by two members, *preGNAI* and *preGNAO,* with *preGNAO* arising after the emergence of Porifera and Ctenophora within Metazoa (Fig. 2, Additional file 2: Supplemental files 1.

More specifically within Deuterostomia, we investigated nine species that diverged before the 2R WGD of Vertebrata, providing a clear starting point before the radiation of this gene family. Within each of these phyla we verified the existence of at least the six established paralogs. Exceptions were found within Urochordata, as we find a lineage-specific loss of *preGNAO* and *preGNAV* at the base of this phylum; this is contrary to previous reports of two *preGNAO* paralogs in *C. intestinalis* [5]. To confirm this lineage-specific loss, we annotated four Urochordata genomes. All four possess multiple *preGNAI*-like genes, but none group within the *preGNAO* subtree (Fig. 2). A putative gene fragment, found only within *B. schlosseri,* groups with *preGNAV* (BS value 66). Due to limited data, it is unclear if this sequence represents a protein-coding gene or a pseudogene (Table 1, Fig. 2, and Additional file 7: Supplemental file 2.

In addition, each phylum interrogated maintained their own number of local gene duplications and/or retrotranspositions for the different primary Gα families (see Appendix A.i for details). To our knowledge, we are the first to report evidence of these duplications and the existence of these retrogenes. Further validation of their presence was interrogated by transcriptome and expression data where available (Additional file 8: Supplemental file 3).

### The (pre) Gαi, q, and v families form a monophyletic group within Gα

**Fig. 3:**
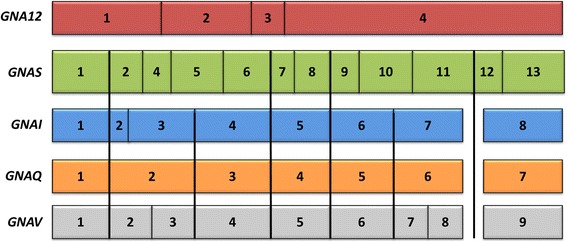
Aligning representative Vertebrata protein-coding exon borders of all five major families of the Gα subunit. The highly conserved exon border positions give insight into the evolutionary divisions of *GNA*- genes. All protein-coding exons are represented as boxes which correlate with the curated average exon size (introns removed). *GNAI* and *GNAQ* share many exon borders positions (black lines) and four split codons (not shown) suggesting a closer evolutionary relationship. *GNAV* also shares six exon border positions with *GNAI* and *GNAQ*; this suggests that Gαv family is related to Gαi and Gαq despite its gene presence in a limited number of species. All three genes share four exon borders positions with *GNAS* (not considering the alternatively spliced exon3 or the extended exon4 of *GNAS* found in Placentalia). The lack of shared exon borders between *GNA12* and the other subfamilies suggests that *GNA12* may have originated as a retro-gene which independently gained introns

We uncovered the evolutionary relationship of the different families by reconstructing phylogenetic trees based on amino acid and nucleotide sequences and by using the conservation of exon-intron structure as a supportive signal of evolution. *preGNAI*, *preGNAQ*, and *preGNAV* share six exon borders and four split codons (codons encoded across two exons) in comparison to the other families suggesting a common origin for these three families (Fig. 3). Only four major exon borders are shared between these three genes and *preGNAS*.

**Fig. 4:**
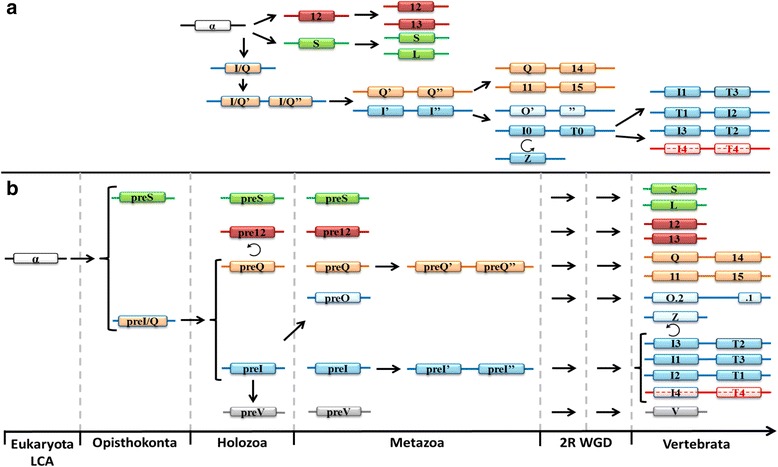
Evolution of the five families of Gα. **a** Summary of previous theories of Gα evolution without relative timelines [3, 6, 7, 9]. An ancestral *GNA* (α-white) underwent a series of duplications before diverging into three primary progenitor families. The progenitor *GNAI/Q* tandemly duplicated before undergoing a larger regional or chromosomal duplication. These gene pairs diverged into *GNAI*-like (blue) and *GNAQ*-like (orange) genes. *GNAS* (green), *GNA12* (red), *GNAQ’*-*GNAQ”*, and *GNAI’*-*GNAI”* all duplicated to give rise to two copies from each parent. *GNAI’*-*GNAI”* duplicated into *GNAO’*-*O″* (ultimately an alternatively spliced gene) and *GNAI0*-*GNAT0* followed by two more duplications of *GNAI0*-*GNAT0*. *GNAZ*, a retrogene of *GNAI0*, was reinserted into the genome before the *GNAI0*-*GNAT0* duplications. **b** New theory of Gα subfamily evolution incorporating current reports [1, 2, 4, 5, 10, 65, 68] with relative timelines included (not fit to scale). A single putative ancestral *preGNA* progenitor (α-white) duplicated into the *preGNAI/Q* progenitor and *preGNAS*. *preGNAI/Q* duplicated into two separate genes that diverged into *preGNAI* and *preGNAQ*. *preGNAV* arose from a duplication of *preGNAI*. *preGNA12* is a retrogene, possibly of *preGNAQ*, though its precise origin is unclear. *preGNAI* later duplicated to give rise to *preGNAO*. Both *preGNAI* and *preGNAQ* underwent independent tandem duplication events before the 2R WGD. *GNAS*, *GNA12* and *GNAQ’*-*GNAQ”* all retained two copies after the 2R WGD, while other hypothetical copies (not shown) were lost immediately after the 2R WGD and are not observed in any extant species. *GNAI’*-*GNAI”* retained three copies of this gene pair after the 2R WGD (*GNAI4* remains only in lampreys). Other, lineage-specific deletions occurred for *GNAV*, *GNAT3*, *GNAI4*, and *GNAT4* as described in the main text. *GNAO* gained alternative splicing of exons 7, 8 after 2R WGD (*O.2*–*.1*). The retrogene *GNAZ* emerged in the Vertebrata lineage from a *GNAI* gene. Lineage-specific duplications and retrogenes are not included for clarity. Straight arrows depict duplications (local, tandem duplications, or WGD), curved arrows depict retrotranspositions. Curated *preGNA*- genes are denoted with “pre-” while “GNA” is removed for clarity in all paralogs. LCA = Last Common Ancestor

Focusing on the Gαi and Gαq families, it was theorized by Wilkie et al. that a progenitor gene to *GNAI* and *GNAQ* (denoted here as *preGNAI/Q*) was tandemly duplicated (*preGNAI/Q’*-*preGNAI/Q”*) and then underwent a larger chromosomal or regional duplication which ultimately led to the *preGNAI’*-*preGNAI”* and *preGNAQ’*-*preGNAQ”* gene pair arrangements [3] (Fig. 4a). Indeed, many others also noted the similar exon-intron organization between paralogs of the Gαi and Gαq families; taken together, this strongly suggests a shared ancestral tandem duplication between these families [6, 10, 66, 67]. Our genomic data of the exon lengths, positions of exon borders, split codons shared across two exons, conserved synteny mapping (gene co-localization) and sequence similarities also support a tandem duplication event and a regional duplication event of a *preGNAI/Q* progenitor. However, we propose that the regional duplication and divergence into two separate genes preceded the two independent tandem duplications.

This hypothesis is supported by the following observations: (1) The individual *preGNAI* and *preGNAQ* genes are encoded by eight and seven protein-coding exons, respectively. The family-specific exon borders are conserved across all paralogs within Cnidaria, Placozoa and Porifera, excluding lineage-specific variations within Protostomia and prior to Parazoa (Fig. 3). (2) *preGNAI* and *preGNAQ* are not arranged in tandem within the investigated Protostomia and non-Bilateria Metazoa species. Taking the evidence of (1) and (2) together, the scenario by Wilkie et al. would require independent intron gain and loss events within exon2/3 of *preGNAI* and exon2 of *preGNAQ* as well as independent lineage-specific losses of one of the gene copies in both *preGNAI’* and *preGNAQ’* gene pairs in the lineages which evolved after the divergence of *preGNAI/Q* into separate genes.

Therefore, we reject the highly unlikely hypothesis of a tandem duplication occurring before the duplication and divergence of *preGNAI* and *preGNAQ* into separate genes [3] and propose that *preGNAI* and *preGNAQ* underwent independent tandem duplications preceding the 2R WGD of Vertebrata. This gave rise to the *preGNAI’*-*preGNAI”* and *preGNAQ’*-*preGNAQ”* paralog pairs that retained their tandem orientation (Fig. 4). These genes are also referred to as *GNAI0*-*GNAT0* and *GNAQ/11*-*GNA14/15*, respectively. Further studies will be required to validate the details of this hypothesis, specifically within non-Metazoa lineages.

No confirmed tandem duplications of *preGNAQ* were found in the investigated species prior to the 2R WGD of Vertebrata suggesting that *preGNAQ* tandemly duplicated into the *preGNAQ’*-*preGNAQ”* pair at the root of the Vertebrata lineage prior to the 2R WGD events. This progenitor pair then duplicated twice and retained the two gene pairs *GNAQ*-*GNA14* and *GNA11*-*GNA15* in Vertebrata.

We identified tandem duplications of *preGNAI* into what could be the progenitor *preGNAI’*-*preGNAI”* arrangements in Placozoa and Hemichordata. The gene pairs are both arranged in head to head orientations similar to those found in the two of the *GNAI* and *GNAT* gene pairs of Vertebrata. The Placozoa *preGNAI* duplications (GIa_Tadhaerens and GIb_Tadhaerens) both group within the *preGNAI* subtree with medium BS values (43). Within Hemichordata, one gene copy (GIa_AcornWorm) groups with the *preGNAI* subtree while the other forms the root of the *GNAT* subtree (GIb_AcornWorm) (Fig. 2). Though this grouping suggests that the gene pair may be a *preGNAI0*-*preGNAT0* set, the low BS value (14) prevents this conclusion. All other identified *preGNAI* duplicates are not in a tandem arrangement; however, their small contig sizes prohibit thorough examination of conserved synteny. Overall, this suggests that the tandem duplication of *preGNAI* could have occurred prior to the emergence of Deuterostomia, but our annotations are not sufficient for further speculation without including more sequences and synteny information.

### Independent duplications of *preGNAI* led to the emergence of *preGNAV* and *preGNAO*

We further expand on the hypothesis set by Wilkie et al. [3] by including Gαv into our analysis. Discovered in 2009 [65] Gαv represents what some suggest is the fifth and final family of the G protein α subunit in animals [68]. We hypothesize that *preGNAV* originated from an ancestral duplication of *preGNAI* within or just prior to the emergence of Holozoa as we and others [5, 65] have found this paralog across Holozoa lineages.

**Fig. 5:**
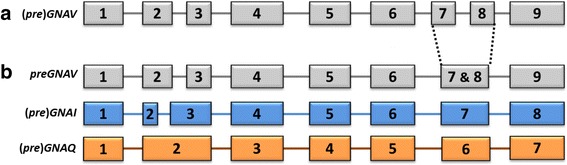
Evolution of Gαv. **a** A schematic representation of the conserved exon-intron structure of Gnathostomata, Cephalochordata, Porifera and Ctenophora (*pre*)*GNAV* genes with 9 protein-coding exons (grey boxes). Box sizes roughly correlate with exon size, while line lengths do not correlate to intron size. **b** The exon-intron structure of Filasterea, Cnidaria, Echinodermata and Hemichordata *preGNAV* genes. This *preGNAV* has no intron to divide exon7 and 8, making its exon-intron structure closely akin to (*pre*) *GNAI* (blue boxes) and (*pre*) *GNAQ* (orange boxes) exon-intron structures. This may represent an ancestral exon-intron structure of *preGNAV*

Gαv has been uniquely identified as a separate family by its exon-intron border positions, sequence motifs, and its position as a separate subtree (Fig. 2); however, its gene structure also provides a link to the Gαi and Gαq families. In comparison to (*pre*) *GNAI* genes, exon7 is split into exon7 and 8 in the *GNAV* of Vertebrata, and intron2 has a different location within the coding sequence (Fig. 5a). From our analysis, we find that the split exon7 and 8 of (*pre*) *GNAV* exists within Ctenophora, Porifera, Cephalochordata and Gnathostomata (jawed vertebrates). Within Filasterea, Cnidaria, Echinodermata and Hemichordata, we find an exon-intron structure of *preGNAV* closely akin to *preGNAI* and *preGNAQ* (Fig. 5b). We and others [5, 65] find no evidence of full-length *GNAV* sequences in the Agnatha (jawless vertebrates), or in any of the four Urochordata species investigated. It is tempting to speculate that the non-split exon structure represents the ancestral exon-intron structure of *preGNAV* while an additional intron was gained in the specific lineages. Intron gains are an unsurprising addition to gene structures, given the usefulness of introns for elevated transcript accumulation, maturation, and splicing of protein-encoding genes [69–74].

Note that the ML gene tree cannot resolve whether *preGNAV* emerged by duplication of *preGNAI/Q*, *preGNAI* or *preGNAQ* as the respective nodes are not well supported (Additional file 9: Figure S7). One of those possibilities is shown in Fig. 4.

### *preGNA12* originated from a Retrotransposition

**Fig. 6:**
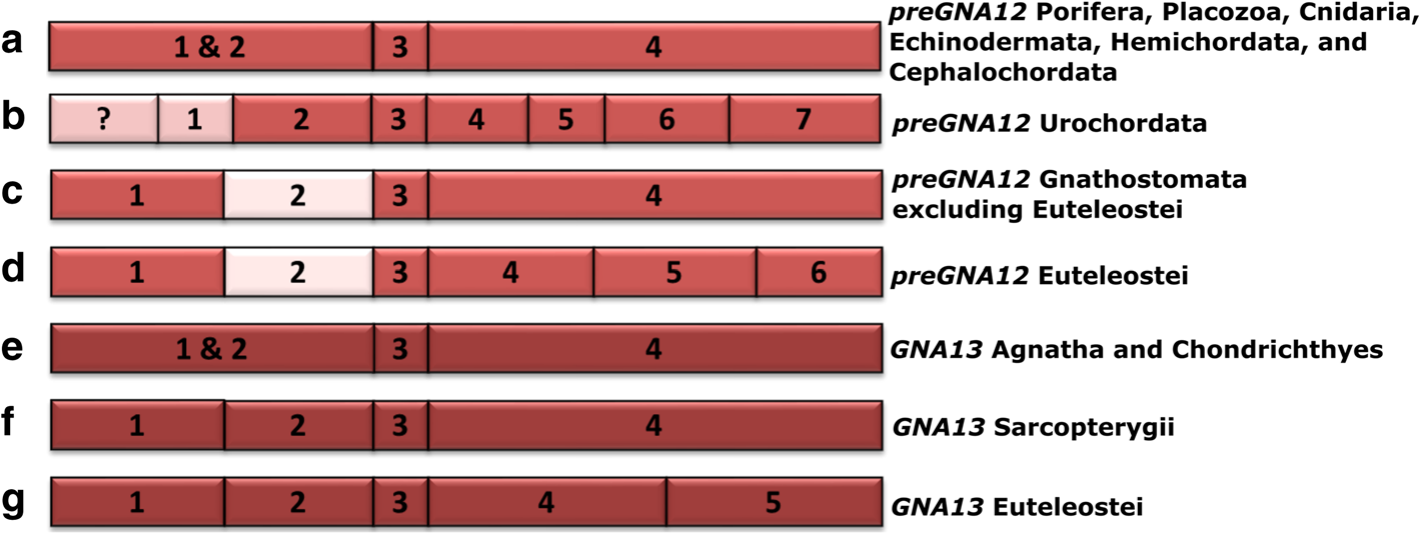
Flexibility of exon-intron borders within the (pre)GNA12 and GNA13 genes. The positions of (pre)GNA12 and GNA13 exon borders (represented boxes) change across phylogeny. Box lengths correlate with average curated exon lengths (introns removed). **a**) preGNA12 (red) has three protein-coding exons in Placozoans, Cnidarians, Echinodermates, Hemichordates, and Cephalochordates. **b**) In Urochordates, the first exon of preGNA12 is divided into at least two exons while the final exon is divided into four exons. As the 5’ sequence is unresolved, more exons may be present (pink with ?). **c**) GNA12 exon-intron structure in jawed vertebrates (excluding euteleosts). The exon sequences upstream of exon3 are not resolved in either jawless vertebrate (lamprey) species investigated. The 5’ end of exon2 is extended by nine nt (pink) in all jawed vertebrates including euteleosts. **d**) GNA12 exon-intron structure in euteleosts (after 3R WGD but not in zebrafish) **e**) GNA13 (dark red) exon-intron structure in jawless vertebrates and cartilaginous fish. GNA13 arose after the 2R WGD that occurred before the emergence of vertebrates. Note that the exon border positions are identical to the GNA12 from (**a**). **f**) GNA13 exon-intron structure in lobe-finned fishes. The exon positions are identical to GNA12 in jawed vertebrates (except euteleosts) (**c**). The GNA13 sequence is extended by one split codon between exon1 and 2 and six nucleotides within exon2 (not shown). **g**) GNA13 exon border positons of euteleosts. The split codon and extended exon2 sequences are maintained

The (*pre*)*GNA12* gene shares no exon border positions or split codons across exons with any of the other members of the Gα family (Fig. 3). Instead, its exon-intron structure hints that *preGNA12* originated from a retrotransposition (Fig. 4b). The ML tree (Fig. 2) suggests *preGNA12* may have originated from a *preGNAQ* sequence, but more sequences are required to interrogate this origin as only one investigated species of non-Metazoa Holozoa possesses *preGNA12* sequences. After the retrotransposition, introns were gained at various positions along the gene within the *(pre)GNA12* family in different branches of Holozoa (Fig. 6a-d).

The same is true after the duplication of *preGNA12* (into *GNA12* and *GNA13*) coinciding with the 2R WGD. The *GNA13* paralog is conserved across Vertebrata, but we see altered exon-intron border positions between species which arose before and after the 3R WGD of Teleostei (Fig. 6e-g) (the 3R WGD is discussed below). Intron gains have been found to promote gene expression, transcript maturity, accumulation, and processing [69–74]. The lack of similarity to the other family members’ exon-intron structures, and its diversity in function [75] suggest the possibility that *preGNA12* underwent neofunctionalization after retrotransposition.

### Gαs is related to Gαi/q

Excluding retrogenes and gene fragments, *preGNA*- genes (*preGNAV*, *preGNAI*, *preGNAQ*, and *preGNAS*) shared at least four exon border positions and three split codons (codons encoded across two exons). This suggests that *preGNAI/Q* and *preGNAS* may have arisen as a result of a gene duplication event from a common ancestor, though exon border information alone is not sufficient to draw this conclusion (Fig. 4). Further analysis is required to ascertain the exact evolutionary relationship between the Gαs and Gαi/q families; however, we see that (*pre*) *GNAV* and (*pre*) *GNAI* form a monophyletic group while (*pre*) *GNAS* clusters outside of this branch on the ML tree (Fig. 2, Additional file 9: Figure S7).

### Individual exon duplications of *preGNAI/Q* and *preGNAS* in Cephalochordata

Prior to the 2R WGD, many paralogs underwent independent, local, single exon duplication events that give rise to alternative splice variants with mutually exclusive exons. Our findings are expanded upon in Appendix A.ii. We found alternative isoforms that arose by exon duplications for preGNAI, preGNAQ, and preGNAS. These may translate into proteins with diverse functions as these alternative transcripts differ in sequence around critical functional and protein-interface regions.

### Gα paralog evolution after the Vertebrata 2R WGD

#### Paralog gains and losses

After a whole genome duplication event, new genetic material will either be maintained (if evolving under purifying or positive selection pressures) or will vanish into the genomic background (if evolving under neutral selection) [76]. Duplicated genes that are maintained may gain new functions or subfunctionalize through mutations in the protein-coding sequence. Temporal and spatial expression patterns may be altered through changes in regulatory regions of the gene. Changes may be maintained to compensate for dosage effects, or serve as a failsafe against the accumulation of deleterious mutations [77–79]. It was estimated that after the 2R WGD of Vertebrata only 20–25% of the duplicated genetic material was retained within genomes [62, 80]. Genes with a low rate of amino acid substitution are more likely to be retained after a WGD [81], as are genes involved in the nervous system [82] or cellular signaling [83].

The Gα subunit is considered a housekeeping gene due to its pivotal role in transducing and amplifying signaling cascades in all cells. Many paralogs are ubiquitously expressed (Gαs, 12, 13, q, i2) in Mammalia tissues, and all but Gα14 and Gα15 are expressed in the brain or neurosensory tissues [75]. Therefore, the duplicated and retained *GNA*- genes (Table 1b) are expected to evolve under strong purifying pressure to prevent the gain of deleterious mutations. Many duplicated Gα paralogs that were retained after the 2R WGD gained new functions, interaction partners, tissue specificity and/or new cellular signaling properties [8, 75].

### The radiation of Gαi

**Fig. 7:**
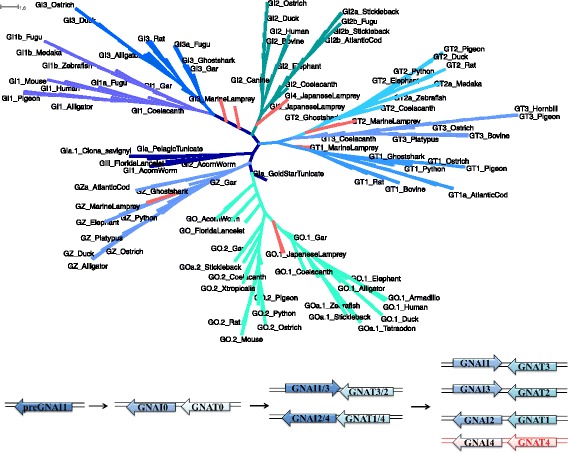
ML tree of the Gαi family resolves gene relationships. **a** ML tree built with all protein-coding sequences found within the (pre) Gαi family (*preGNAI*, *GNAI1*–*4*, *GNAT1*–*3*, (*pre*) *GNAO*, and *GNAZ*) in all Deuterostomia lineages evaluated. All lamprey branches are denoted in pink. The outgroups of the *GNAI* and *GNAT* genes have high bootstrap supports (*GNAT1*–100 and *GNAI2*–92). The two *GNAT* lamprey genes form a monophyletic group with *GNAT1* and *GNAT2* of jawed Vertebrata, as do *GNAO* and *GNAZ* lamprey genes with their respective subtrees. **b** *GNAI1* and *GNAI3* are situated next to *GNAT2* and *GNAT3*, respectively, within the genome. These pairs are the result of the duplication of the respective GNAI1/3-GNAT3/2 gene pair. *GNAI2* and *GNAI4* are situated next to *GNAT1* and *GNAT4*, respectively; they arose from GNAI2/4-GNAT1/4 gene pair during the same duplication event. *GNAI4* and *GNAT4* (red) are not observed in the genomic data investigated (except for *GNAI4* in lamprey)

The Gαi family expanded in Vertebrata to include *GNAI1*–*4*, *GNAT1*–*4*, and *GNAZ*, in addition to *GNAO*. *GNAT4* and *GNAI4* were quickly deleted. A ML tree built on the nucleotide level further supports the emergence of these paralogs from the 2R WGD in Vertebrata, and shows the pattern of *GNAI0*-*GNAT0* duplication by resolving *GNAI2* as the outgroup of the Gαi subfamily and *GNAT1* as outgroup of the Gαt subfamily when excluding lamprey sequences (Fig. 7, Additional file 3: Supplemental file 4). These outgroups support the hypothesis of the individual Gαi and Gαt subfamily members emerging through the tandem duplication of *preGNAI* followed by two consecutive whole genome duplications. The tree constructed in the current study has a different tree topology than those constructed with amino acid sequences by Lagman et al. [8] and Krishnan et al. [5]. This tree topology is in accordance with the arrangement of *GNAI2* and *GNAT1* as neighbors, which resolves the inconclusiveness of previous studies.

We found no evidence of the proposed *GNAT*-like progenitor gene [9] in the Chordata lineage (*preGNAT0*) prior to Vertebrata divergence; this is in accordance with previous findings [8]. In addition, we identified a putative *preGNAT0* sequence within the Hemichordata lineage (denoted GIb_AcornWorm), that is positioned in a head to tail arrangement with a *preGNAI* gene (GIa_AcornWorm). It is not clear, whether this sequence represents a 1:1 ortholog to *GNAT0* due to a low BS support (14) of GIb_AcornWorm with the split of the Vertebrata *GNAT* subtree.

*GNAT3*, which is situated adjacent to *GNAI1* in a head to head orientation within Vertebrata genomes, is lost in a lineage-specific manner in Amphibia and Actinopterygii as reported previously [6, 10] and confirmed by the current study. The conserved syntenic regions around *GNAI1* are maintained, revealing that this loss of *GNAT3* is local and not connected to additional rearrangements. The fourth *GNAI*-*GNAT* gene pair (*GNAI4*-*GNAT4*) was predicted to be immediately lost subsequent to the 2R WGD [7]; synteny mapping in humans show a conserved fourth set of genes surrounding the region where the *GNAI4*-*GNAT4* pair was initially situated after duplication and then presumably deleted [7].

However, we found nucleotide sequence evidence for four paralogs of *GNAI* in the Agnatha lineage in both lamprey species investigated, which may correspond to the four copies originating from duplications of the *GNAI0*-*GNAT0* gene pair. All four *GNAI* genes have the same eight protein-coding exon structure with conserved border positions, and the amino acid ML tree shows the putative *GNAI1*–*4* all clustering close to the root of the Gnathostomata *GNAI* subtree (Fig. 2). The nucleotide ML tree provides better resolution with lamprey *GNAT1* and *GNAT2* clustering with their putative Vertebrata 1:1 orthologs (Fig. 7). Synteny mapping supports the expected head to tail orientation of the *GNAT1*-*GNAI2* pair and the head to head orientation of *GNAI3*-*GNAT2*. In addition, *GNAI1* synteny supports the loss of *GNAT3* by maintaining conserved flanking gene neighbors. While a fourth copy of *GNAI* (*GNAI4*) has been briefly described previously in lampreys [10], the lack of clear synteny information prevents further validation of its origin in the Vertebrata ancestor. Though the conservation of exon border positions, split codons, and nucleotide sequence support the assignment of *GNAI4* to the Gαi subfamily, evidence of conserved gene neighbors are needed to ascertain if this paralog is the product of an independent duplication or if it is a product of the 2R WGD. There is no evidence of 1:1 orthologs to the lamprey-specific *GNAI4* in other Vertebrata lineages. We also reveal that the putative fourth member of *GNAT* proposed by [9] is rather a putative *GNAT1* ortholog considering synteny information and ML tree topology, not a novel *GNAT* gene or the missing fourth member.

One significant improvement from our study comes from the inclusion of two Agnatha species. The genome of *P. marinus* used in previous studies is highly fragmented preventing reconstruction of complete gene sequences or evaluation of synteny information. Including an additional species allowed us to clarify ambiguities present in those regions. Nevertheless, we cannot resolve whether lamprey *GNAI1*–*3* and *GNAT1*–*3* represent 1:1 orthologs to human *GNAI1*–*3* and *GNAT1*–*3*, respectively, despite the conserved tandem orientation of the genes and conserved synteny around several of the paralogs, as the position in the ML tree is not well supported and partially conflicting. The lamprey Gαq family members are also situated near the root of the Q/11 or the whole Q family subtree in the ML tree (see below). This reflects the current debate about the exact timing of the 2R WGD relative to the divergence of lampreys and possible lamprey-specific (whole) genome duplications [13, 84].

### Gαz

We identified full-length *GNAZ* genes in all Vertebrata species evaluated (including ghostshark), as well as partial genes (due to small contig size) in both lamprey species - contrary to previous reports [5], Contrary to previous theories [6], we found no substantial evidence of *preGNAZ*-like sequences in non-Vertebrata Deuterostomia. The ML tree composed of all five primary families (Fig. 2) shows *GNAZ* grouping tightly within the Gαi family; taken together, this suggests *GNAZ* originated from a duplication of a Gαi family member in early Vertebrata evolution.

Two *preGNA*- sequences (*B. schlosseri* and *T. adhaerens*) are seen on the ML tree to group with the *GNAZ* branch, albeit with low bootstrap values (32). Both genes in question possess a gene structure that is highly similar to the eight exons of *preGNAI and* are thus excluded as 1:1 orthologs of a putative *preGNAZ*.

The exon-intron structure of *GNAZ* largely deviates from the exon-intron structure of other Gαi family members (Additional file 10: Figure S2). *GNAZ* is located on the opposite strand within an intron of the *RSPH14* gene. We hypothesize that *GNAZ* emerged through a retrotransposition into this position and subsequently gained one intron. This resulted in the conserved two protein-coding exon gene structure. Appendix B.i discusses further analysis done to investigate whether the intron of *GNAZ* carries signatures of insertion mediated by a retrotransposon mechanism; however, no conservation of these residues was found.

### Gαo

**Fig. 8:**
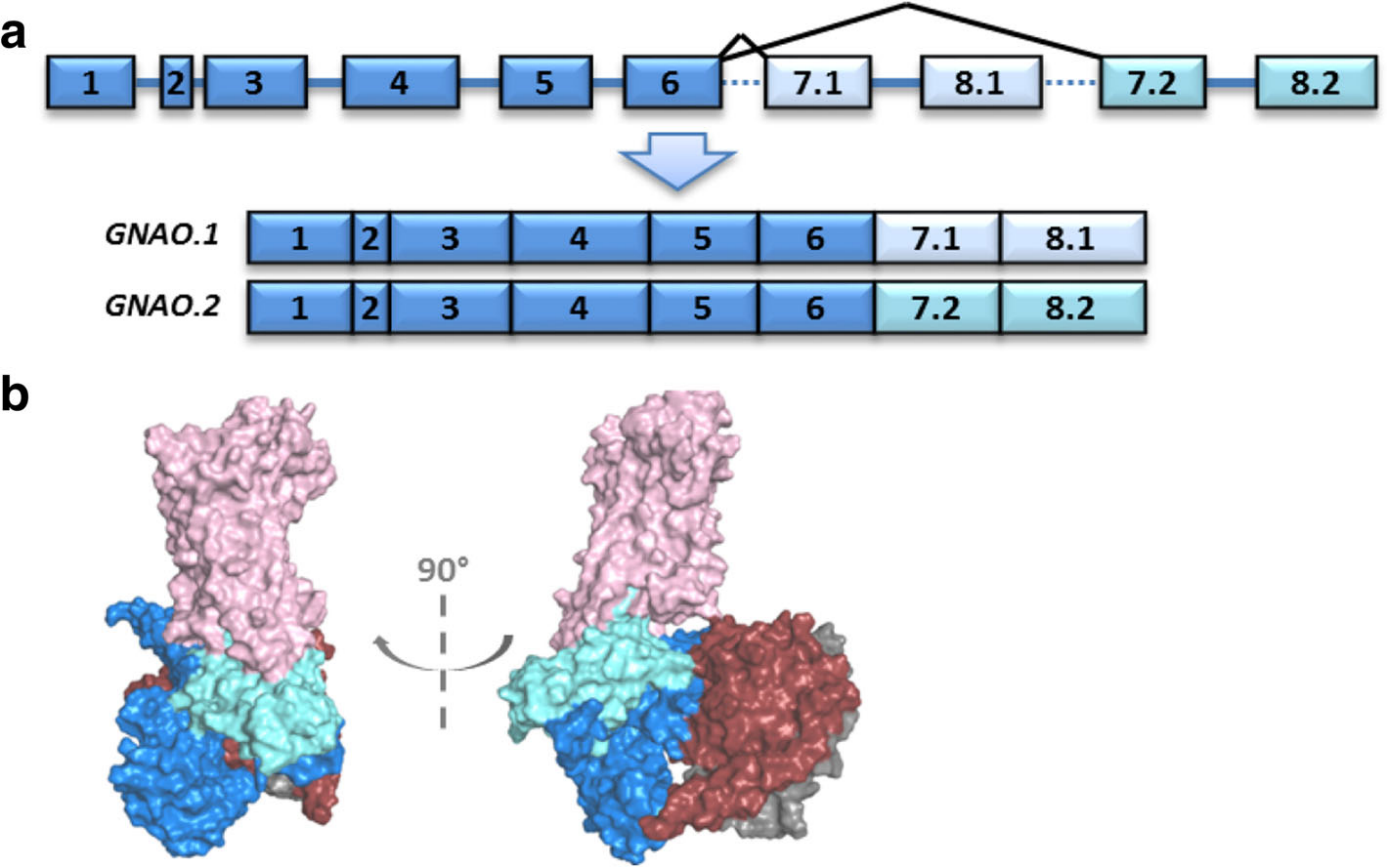
Alternative Splicing of *GNAO*. **a** The Vertebrata *GNAO* gene has two transcripts (.1 light blue and .2 cyan) that arise from mutually exclusive splicing of its final exon pair: exon7 and 8. Note that exon lengths correlate with box lengths while lines do not correlate with intron size. **b** Tertiary structural model of the heterotrimeric G protein. Gαo (blue) and the heterotrimer Gβγ subunits (crimson/grey) coupled to a GPCR (pink). The two mutually exclusive exons encode regions necessary for coupling to active GPCRs and subsequently activating the G protein itself. The differences in sequences may influence coupling affinity and activation efficiency

Though *preGNAO* emerged before the 2R WGD, we do not find evidence of additional *GNAO* gene copies being retained in Vertebrata after the whole genome duplications (with the exception of Teleostei after the 3R WGD, discussed below). Instead we observe a local duplication that gave rise to two mutually exclusive exons (7.2–8.2 and 7.1–8.1) that are conserved in all major Vertebrata clades (Fig. 8a).

**Fig. 9:**
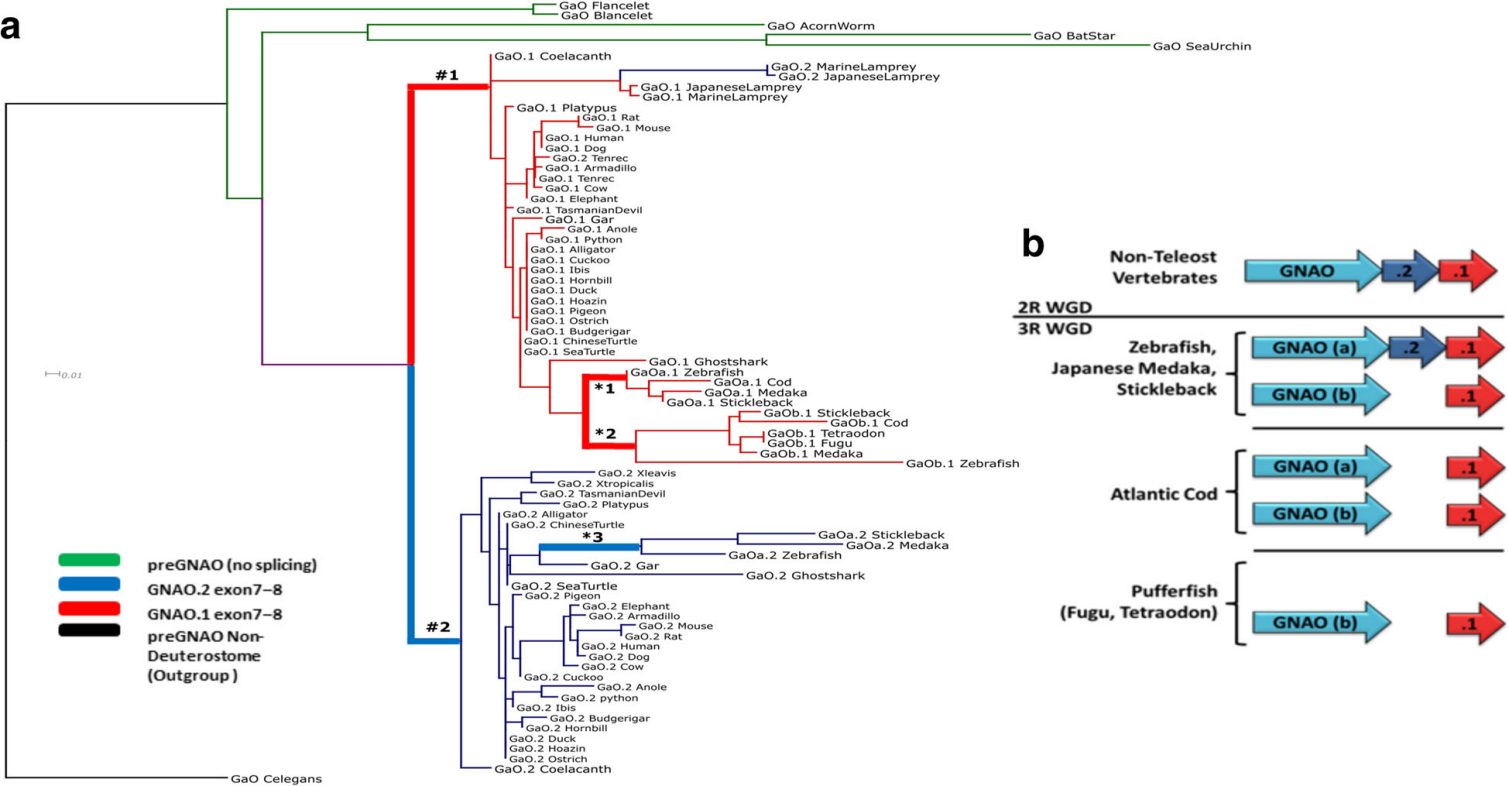
Retained exons of *GNAO* after 3R WGD in Teleostei. **a** A ML tree of exon7 and 8 nucleotide sequence indicates which exon pairs were retained across different Teleostei. Branches tested for positive selection are marked by ‘#’ and ‘*’. **b** After the 3R WGD, only one gene copy of *GNAO* (named copy ‘a’) maintained two sets of the mutually exclusive exon7–8 endings (variant ‘.2’ – blue, variant ‘.1’ – red). In Atlantic cod, both gene copies possess only one set of the final exons which was identified as the ‘.1’ variant. In both species of pufferfish, only the ‘b’ copy of *GNAO* was retained with the ‘.1’ exon variant

The resulting two Gαo isoforms likely show functional differences as the final two exons of *GNAO* map to regions of the tertiary Gαo protein structure (Fig. 8b) which have been shown to be necessary for receptor-G protein interaction [85, 86], receptor selectivity, and subsequent G protein activation [58, 87–89]. *GNAO.1* evolved slightly faster after the duplication in comparison to *GNAO.2* as indicated by a longer ancestral branch (Fig. 9). This is in accordance with results from the natural selection analysis. This points to signs of positive selection (wFG = 613 +/− 428) acting on roughly 10% of the residues on the *GNAO.1* branch after duplication (1-p0-p1, see Additional file 11: Table S2). Given this small percentage of residues, the exact estimate of selection pressure, ‘w’, in the foreground branch is uncertain. In addition, 88% (+/− 9.9%) of all residues are under strong purifying selection (w0 = 0.017 +/− 0.004). Ten residues which were identified to be positively selected differ systematically between *GNAO.1* and *GNAO*.2; the amino acids are conserved in *GNAO.2* in comparison to the non-Vertebrata Deuterostomia *preGNAO* (Additional file 11: Table S2, Additional file 12: Table S3, and Additional file 13: Figure S3).

### Gαq

Three of the four known family members (prior to Gαv discovery) were previously predicted to be situated on large blocks of duplicated genetic material [6]. We systematically validated that *preGNAQ* duplicates (*GNAQ*, *14*, *11* and *15*) were present in all Vertebrata. The head to tail arrangement of the gene pairs *GNAQ*-*GNA14* and *GNA11*-*GNA15* is conserved in all investigated species. As seen in the ML trees, *GNAQ* and *GNA11* are very closely related while *GNA14* and *GNA15* though diverged, group together.

*GNA14* and *15* have gained sequence divergence, tissue expression specificity and new functionality, while *GNAQ* and *11* appear to be ubiquitously expressed in Mammalia tissues and are involved in a high level of redundant cellular signaling processes [75]. We see two lineage-specific losses of *GNA15* in Coelacanthiformes as well as in Neoaves (supported by loss in all six investigated neoavian species), that are further supported by synteny information, EST and TSA data (Additional file 8: Supplemental file 3).

### Gαs

During the 2R WGD, *preGNAS* duplicated to give rise to *GNAS* and *GNAL* (Gαolf) [6]; *GNAL* developed tissue-specific expression and functional specificity within the olfactory bulb and various neuronal tissues [75]. We found a species-specific loss of *GNAL* in the genome of the green anole lizard. However, when validating this putative loss with transcriptome and expression data, we found evidence of *GNAL* expression within lizard TSA and EST data [17, 21] (Additional file 8: Supplemental file 3c-d). We thus conclude that *GNAL* must be encoded within the genome of the green anole lizard though it is not represented within the investigated genome assembly. Such issues have been previously reported and may be due to problems during scaffold assembly and coverage during sequencing [90].

**Fig. 10:**
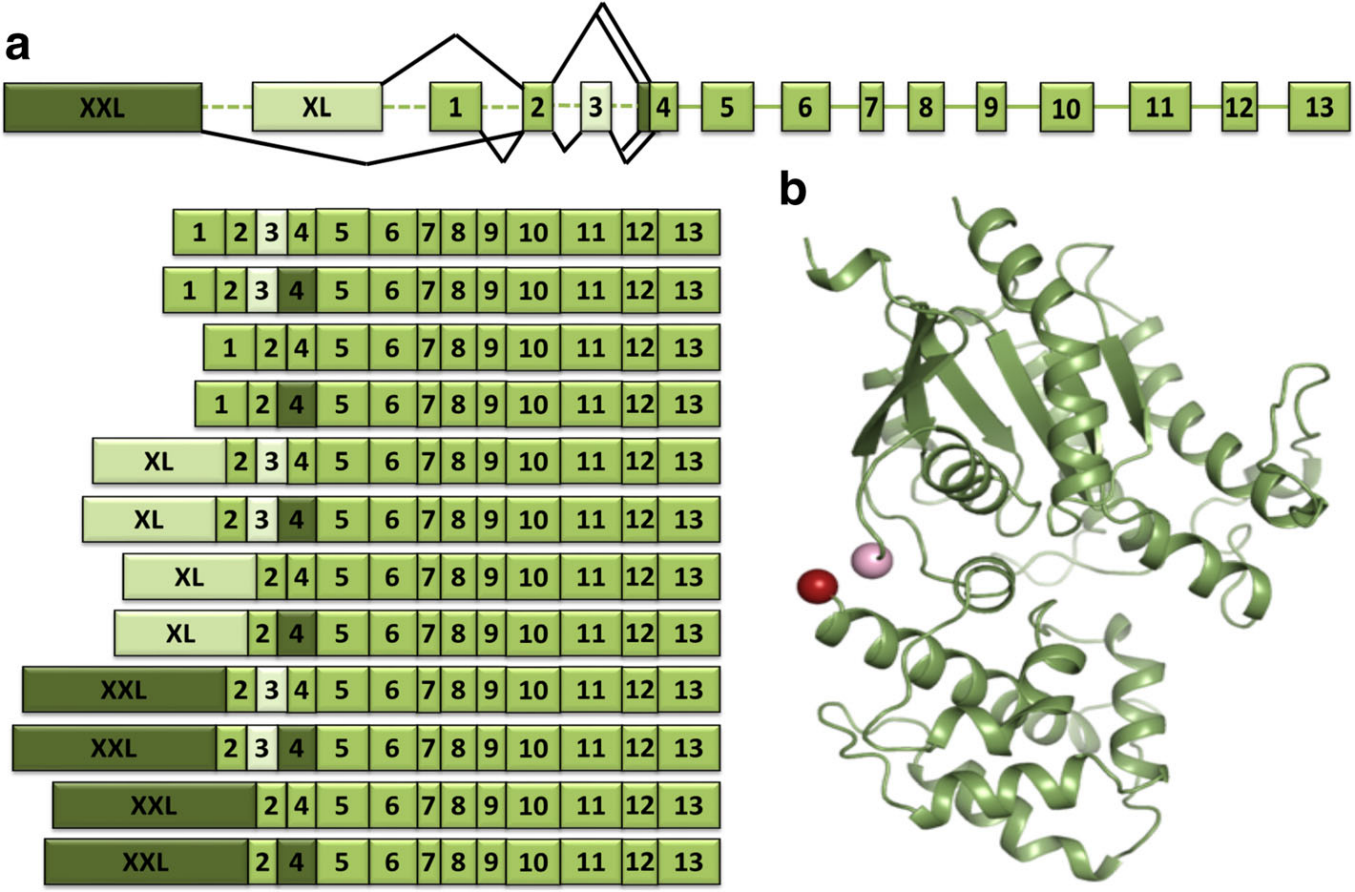
Multiple transcripts are possible from the complex locus of *GNAS*. **a** Different mRNA transcripts can be produced from the *GNAS* locus through alternative splicing. The XXL-exon, though not examined herein, can be alternatively included into the transcript in exchange for exon1 or the XL-exon. In addition, Placentalia possess a cassette exon3 (light green) which can be included or excluded within the transcript; a non-canonical SS can also give rise to an extended exon4 (dark green) in the same species. Box lengths correlate with average curated exon lengths (intron lines do not). **b** Crystallographic tertiary structure of Mammalia Gαs (PDB ID 1AZT [55]) missing exon3. The C-terminus of exon2 (pink sphere) and N-terminus of exon4 (red sphere) are shown

In all investigated Vertebrata genomes, we show that *GNAS* possesses an upstream alternative first exon, extra-long exon (XL-exon) (Fig. 10a), which is similar in sequence to the 3′ sequence of exon1 [91]. *GNAL* also possesses a homologous alternative, longer upstream exon, suggesting that this alternative exon sequence existed before the 2R WGD. The XL-exon appears to be absent in non-Vertebrata Deuterostomia. Nevertheless, we are careful to speculate about the exact timing of its emergence due to 1) the extensive variability in XL-exon’s length and its 5′ sequence which make homology searches challenging, 2) the highly fragmented quality of the non-Vertebrata genome assemblies utilized which hinder even highly refined searches with the EMS pipeline. We were unable to confirm the presence or absence of the XL-exon in *preGNAS* before the 2R WGD based on both genomic and expression data.

In addition to the XL-exon, an extra-extra-long exon (XXL-exon) has been reported upstream of *GNAS* in human and rodent species [92]. Due to its variability in size (approximately ranging from 1400 nt to 2300 nt) and vast sequence divergence, the XXL-exon was not investigated here. Conservation of imprinting [93, 94] and the gene promoter, which is shared with four other upstream genes [95, 96], were not the subject of this study. For excellent reports on the complex *GNAS* gene structure in Mammalia, please see [92, 97, 98].

As another peculiarity, *GNAS* possesses a cassette exon, exon3, which can be skipped during splicing [99, 100] (Fig. 10a). The inclusion of exon3 adds 15 AA to the Gαs protein (14 AA encoded by this exon plus one AA encoded by a split codon shared with exon4). When mapped onto the tertiary protein structure, the amino acid region encoded by exon3, extends a flexible linker between α-helix1 of the enzymatic GTPase domain and α-helixA of the helical domain (Fig. 10b). This region may be important for G protein activation and nucleotide exchange [89, 101].

The cassette exon3 of *GNAS* appears to be a very “recent” evolutionary invention as we only find it conserved in Placentalia (placental mammals) but not in other Vertebrata. Interrogation of available transcriptome and expression data confirmed that there is no evidence of exon3 existence outside of this branch (Additional file 8: Supplemental file 3). The intron between exon2 and 4 is large (~ 43,000–72,000 nt) in non-placental Sarcopterygii, while the homologous region becomes much smaller (~ 6000–9000 nt) after emergence of exon3.

We searched for sequences similar to exon3 in other species of Mammalia to elucidate the possible origin of this new exon. We could not find sequence similarity to human proteins from UniProt KB [40] or the NCBI database [17] or to the intronic region between exon2 and exon4 in 14 Sarcopterygii (lobed-finned fishes) when querying with the amino acid and nucleotide sequence of exon3, respectively. Within Placentalia, a highly conserved sequence stretch of roughly 75 nt is situated upstream and 25 nt downstream of exon3, bookending the exon (Additional file 14: Figure S4). Appendix B.ii discusses predicted motifs for DNA-binding proteins (DBPs) and RNA-binding proteins (RBPs) we identified which may be present within this sequence stretch.

The emergence of exon3 in Placentalia also co-occurs with the ability of exon4 to be extended by three nucleotides (Fig. 10). This extension is mediated by a well-documented non-canonical SS ‘TG’ situated 3 nt upstream of the canonical SS ‘AG’ [100]. The ‘TG’ splice recognition pattern shifts the SS to allow the nucleotides ‘CAG’ to be included within the exon giving rise to four different isoforms around this exon junction variation: exon2-E-exon3-G-exon4, exon2-E-exon3-GS-exon4, exon2-D-exon4, exon2-DS-exon4.

We found no evidence of an extended exon4 outside of Placentalia in any genome interrogated. Therefore, we conclude that exon3 and the extension of exon4 co-occurred in the ancestor of Placentalia after the split from Marsupialia (marsupials). The expression of all four possible variations of transcripts with the inclusion/exclusion of exon3 and the possible extension of exon4 is supported by transcriptome and expression data.

Pyne et al. speculated that the additional amino acid arisen from the exon extension could promote phosphorylation [102]. We did not find any evidence for posttranslational modifications at this or neighboring positions in UniProt KB [40] or the PhosphoSite database [103]. Amino acids encoded by exon3 and the exon4 extension are situated in a flexible linker region between the GTPase domain and the helical domain of the G protein. This region is unresolved in all crystal structures of the Gαs subunit (Fig. 10b).

### Gα12

*preGNA12* was duplicated to give rise to *GNA12* and *GNA13* in Vertebrata during the 2R WGD. Both paralogs are present in all Vertebrata genomes investigated except for Amphibia (*X. tropicalis* and *X. laevis*). Genomic information and available EST data support a loss of *GNA12* (Additional file 8: Supplemental file 3) though *GNA13* is present in both species. Refer to Fig. 6 for altered exon border information.

### Gαv

*GNAV* was the most recently discovered member of the *GNA*- genes [65] due to the widespread loss of this paralog. *GNAV* was lost independently twice within Vertebrata: at the base of Tetrapoda and at the base of Agnatha. Any *preGNAV* gene duplications were not retained after the 2R WGD. Prior to the 2R WGD, *preGNAV* gained an intron dividing exon7 into two (Fig. 5a). This gene structure is maintained in all species of Vertebrata where the paralog is present (ghostshark, coelacanth, gar and Teleostei).

### Retrogenes in Primates

We find that members of four of the five Gα families have been subjected to repeated retrotransposition during very recent evolutionary history, specifically during the evolution of Primates and suborders within (Additional file 6: Figure S1). Eight of the 15 retrotranspositions are species-specific and limited to the marmoset and tarsier-lineages (Additional file 15: Table S4). This might reflect the excess of retrocopies in Platyrrhini (New World monkey) in comparison to Cercopithecidae (Old World monkey) [104]. Additionally, the *GNA11* retrogene *GS1-124 K5.9* was tandemly duplicated twice as indicated by the location of these retrogenes in proximity to their parent retrogene. Surprisingly, the gorilla-specific copy of *GS1-124 K5.9* conserves more than 80% of the full-length open reading frames (ORFs) of the parent gene with 99.34% sequence identity to the protein sequence, although we did not detect any expression. Contrarily, the Cercopithecidae-specific *GS1-124 K5.9* copy is expressed in baboon frontal cortex.

Most of the Primates retrogenes degraded into pseudo-retrogenes conserving several short ORFs that are still similar to the parent genes. Those pseudo-retrogenes are only lowly transcribed in one species in at most two independent RNA-seq experiments considered (Additional file 15: Table S4).

Contrarily, *GS1-124 K5.9* and *GNAQP1* are interesting examples of retrogenes that are functional in several Primate species. We consider both genes to be functional as 1) they conserve a homologous region longer than 40 AA with high similarity to the parent protein across all Catarrhini; 2) promotors are annotated directly upstream on the same strand in human (Ensembl v87 [15]); 3) transcription of both genes in human is supported by the psiCube data as well as by six independent RNA-seq studies retrieved from the Expression atlas [44, 45] (three shown) and by at least one RNA-seq experiment for another Primate species, vervet-AGM and macaque, respectively (Additional file 16: Figure S5, Additional file 15: Table S4). *GNAQP1* is expressed in a variety of tissues, while transcription of *GS1-124 K5.9* was detected in only three tissues in human (testis, choroid plexus, and forebrain Additional file 16: Figure S5 a, c & e). Five independent studies support the expression of both genes in human testis (see Additional file 16: Figure S5e for sixth study) in accordance with the tendency of retrogene expression in testis reported previously [104, 105]. Interestingly, macaque also expresses *GNAQP1* in testis (Additional file 16: Figure S5b).

Two other retrogenes, *AC010975.2* and *RP11-100 N3.2*, are transcribed in human and at least one other species implying that those genes might also be functional, although we detected no conserved ORF or upstream promotor. The *GNA13* pseudogene *AC010975.2* is expressed in human, vervet-AGM and baboon with overlapping tissue expression in pituitary gland across both Cercopithecidae species, while *RP11-100 N3.2* is expressed in human and macaque (not shown). We note that the expression levels found of all (putative) functional *GNA*- retrogenes are in general lower than expression of the parent genes.

The Gα subunit belongs to the fold clan of P-loop NTPases. This clan is one of the few examples of gene families that are consistently highly duplicated via retrotransposition in the different lineages of worm, human and fly [45]. Our observation in this context is in accordance with findings that correlate retrotransposition with the expression level of the parent gene in germ line tissue [106, 107]. Most members of the Gα family are housekeeping proteins that are known to have widely distributed or ubiquitous expression patterns throughout the body [75]. The excess of *GNA*- retrotransposition in Primates likely reflects the known high activity of retrotransposable elements in this clade [105]. (Pseudo-)retrogenes are a potential source for the emergence of paralogs, (long) non-coding RNAs and ORFs encoding small peptides and are often lineage-specific [108]. The latter two types do not necessarily have sequence similarity to the parent protein and can gain functions in a completely different cellular context. In this study about *GNA*- gene and protein evolution, we focused on retrogenes that still show sequence similarity to the parent protein and well annotated human *GNA*- retrogenes. Our retrogene counts thus represent a lower boundary of retrotransposition events. Instead of providing exact counts, we exemplified the high frequency of retrotranspositions in the evolutionary history of *GNA*- genes in the Primates lineage.

### Individual exon duplications in *GNAQ*, *GNA11,* and *preGNAI*

We found additional duplications of exon4 in *GNAQ* and *GNA11* in some species of Vertebrata. Surprisingly, the homologous sequence of *preGNAI*, encoded by exon5, can also be alternatively spliced in Urochordata. The sequence diversity in the alternatively spliced transcripts may have an important role in providing novel functionality as these sequence regions correspond to important interface regions within the protein tertiary structure. For further analysis of these exons, please see Appendix B.iii, Additional file 17: Figure S6, and Additional file 4: Supplemental file 5.

### Non-canonical splice sites of *GNAI1*

We found conservation of canonical ‘GT-AG’ splicing patterns for all of the exon sequences annotated with two exceptions. The first is the alternative upstream splice site (SS) of exon4 in *GNAS* in Placentalia which has been discussed above. The second is the highly conserved 5′ non-canonical SS ‘GC’ in intron6 of *GNAI1* in most species of Sauropsida and Mammalia (Additional file 9: Figure S7). This non-canonical splice site co-occurs with an extension of the consensus motif within the surrounding exonic and intronic regions. As the switch from canonical to non-canonical SS, and its subsequent systematic conservation, is surprising, we evaluated possible selective pressures within this region. Our analysis of motifs for DNA-/RNA-binding proteins (DBPs/RBPs) is detailed in Appendix B.iv and Additional file 9: Figure. S7 and Additional file 18: Figure S8.

### Gα paralogs after the 3R WGD in Teleostei

#### Paralog gains and losses

In addition to the Vertebrata 2R WGD [61, 62] a third round of whole genome duplication (3R WGD) occurred at the base of Teleostei [64, 109, 110]. It is estimated that over 75% of the genes which arose from the 3R WGD were subsequently lost [109, 110]. The paralog gains and losses obtained from the EMS are summarized in Table 1. We confirmed and updated the paralog counts reported by Oka et al. [10]. Briefly, we find two copies of *GNAI1*, *GNAI2*, *GNAL*, *GNA11*, and *GNA14* in all Teleostei. *GNAV*, *GNAS*, *GNAQ* all have two copies present in Euteleostei, but only one copy remains in zebrafish. *GNAO* and *GNA13* also have two copies, though there are lineage-specific deletions in pufferfish and Atlantic cod, respectively. Only one copy is maintained after the 3R WGD for *GNAI3*, *GNAZ*, *GNAT1*, and *GNAT2*. *GNA12* also has one copy retained in Euteleostei, but two copies are present in zebrafish. It appears that zebrafish *GNA15* underwent several duplications resulting in an arrangement of four *GNA15* paralogs [10] situated on the same chromosome next to each other with otherwise conserved synteny. At least three of the four copies are expressed as confirmed by EST and TSA data. *GNAT3* is deleted in all Actinopterygii. Of the paralogs that are retained, we find variations in the positions of intron-exon borders (*GNA12* and *GNA13*) and variations in alternative splicing patterns (*GNAO*, *GNA11*, *GNAQ*) as discussed in other sections.

### *GNAO* alternative splicing in Teleostei

Two copies of *GNAO* were retained after the 3R WGD (except within Tetraodontidae -pufferfish). In zebrafish, medaka and stickleback both mutually exclusive exons (exon7.2–8.2 and exon7.1–8.1) were retained in one copy (referred to as gene copy ‘*a*’ - *GNAOa.1* and *GNAOa.2*). The other gene copy (*GNAOb*) lost one pair of exons7–8 immediately following the 3R WGD. In Tetraodontidae, we see a lineage-specific deletion of the complete *GNAOa* copy (Fig. 9a).

To determine which copies of the exon sequences were retained in these paralogs (either variant .1 or .2), we created a ML tree of the nucleotide sequences for *GNAO*’s exon7 and exon8 across all phylogenetic branches evaluated. We see that the alternatively spliced exons7 and 8 of *GNAOa* possess both the .1 and the .2 transcript variants while all of the .1 sequence variants are conserved within *GNAOb*. Thus, we resolve that the .2 exon pair of *GNAOb* was lost at the base of Teleostei and that *GNAOa.2* was lost independently in *G. morhua* (Atlantic cod). In our selection analysis, we did not detect any residues under positive selection in any of the ancestral branches tested (*GNAOb.1*, *GNAOa.1* and *GNAOa.2*). While all residues of exons 7.1 and 8.1 are under strong purifying selection in both ‘a’ and ‘b’ copies (w = 0.0075), the selection pressure is slightly released with about 6% of residues evolving under neutral selection in the ancestral branch leading to *GNAOa.2*. This might also reflect the released pressure that ultimately led to the loss of *GNAOb.2* in all Teleostei.

## Conclusions

The strength of this study comes from the inclusion and curation of genes from highly fragmented genome assemblies in addition to the genomes of well-studied model organisms. Despite improved long-read genome sequencing techniques, computational assembly of accurate whole genome sequences remains a challenge [11]. High sequence similarity between genes due to homology remains challenging when assembling DNA-seq reads into larger scaffolds or when mapping RNA-seq reads to a genome. The ambiguity of these regions can result in chimeric gene annotations where two different genes are presumed to be one. Additional errors can be introduced via automated gene prediction tools which probe the assembly. For a more thorough examination of these hurdles please see [11, 24].

The ExonMatchSolver (EMS) algorithm [11] was developed to assist in overcoming some of these challenges when curating highly fragmented genome assemblies. EMS differs from other methodologies by querying for the collective “match” of all paralogous genes of a protein family within an individual genome assembly. As the family of heterotrimeric G proteins contains many paralogs, we used the EMS technique to annotate and disambiguate paralogs of the Gα subunit across phylogeny. Despite its usefulness, it is of note that the EMS pipeline does not resolve inversions of exons or significantly altered exon-intron structures. Instead this tool provides contexts for manually resolving such ambiguities in the nucleotide sequences.

Through the use of the EMS pipeline to assist in the curation of the *GNA*- genes across a dense species sampling, we have identified dozens of sequence deviations and inconsistencies within the examined species and paralogs compared to previous works and genome annotations. In this work, we have uncovered many paralogs of *GNA*- not identified by previous methodologies; this is likely due to the use of coarse-grained approaches which misidentified the presence and absence of genes and/or due to the reliance on gene trees covering a limited range of species. Our updated report allows us to refine the theories surrounding Gα evolution.

In addition to the major findings of gains and loss events and paralog family assignments within this manuscript, we also uncovered previously unknown variance in gene duplications, the conservation of alternative splicing patterns, exon duplications/insertions, non-canonical SS, conserved DBP and RBP motifs, and traced back the emergence of Primate retrogenes. Each of these variants are expanded upon in the appendices. In addition, our curated sequences have been made available for use as the basis of future annotations, sequencing efforts, and as seed inputs for developing biological questions surrounding the Gα family.

### Additional files

Additional file 1: **Table S1.** Species Evaluated. All major branches of Deuterostomia were investigated using the EMS pipeline (where sequenced genomes exist). Nine species were also included from non-Deuterostomia Opisthokonta lineages to act as outgroups. Column1 – Description of phylogenetic branch. Column2 – Common name (*Genus species*). Column3 – Genome assembly used. Column4 – Accession number for genome assembly, when available. (NEXML 673 kb)

Additional file 2: **Supplemental file 1.** Maximum Likelihood Tree of (pre)GNA- genes. ML tree built with all paralogs and sequences evaluated in Nexml format. Bootstrapped replicates were summarized into Extended Majority Rule Consensus Trees and reported with bootstrap (BS) values. (PDF 4751 kb)

Additional file 3: **Supplemental file 4.** Maximum Likelihood Tree built on the nucleotide level further supports the emergence of GNAI1–4, GNAT1–4, and GNAZ, in addition to GNAO paralogs from the 2R WGD in Vertebrata. It shows the pattern of GNAI0-GNAT0 duplication by resolving GNAI2 as the outgroup of the Gαi subfamily and GNAT1 as outgroup of the Gαt subfamily when excluding lamprey sequences. (NEXML 302 kb) 

Additional file 4: **Supplemental file 5.** Maximum Likelihood Tree of duplications of exon4 in GNAQ and GNA11 and the homologous sequence of preGNAI, encoded by exon5, duplicated in Urochordata. Bootstrapped replicates were summarized into Extended Majority Rule Consensus Trees and reported with bootstrap (BS) values in Nexml format. (NEXML 74 kb)

Additional file 5: **Supplemental file 6.** ML trees of the non-Vertebrata Deuterostomia preGNAI and preGNAQ nucleotide sequences which corresponded to the mutually exclusive exons, exon6 or exon5, respectively. Bootstrapped replicates were summarized into Extended Majority Rule Consensus Trees and reported with bootstrap (BS) values in Nexml format. (NEXML 37 kb)

Additional file 6: **Figure S1.** Primates species investigated for retrogenes. The existence of *GNA*- pseudogenes was investigated within human and 11 other Primates species. A) Primates species investigated. The Latin names and clades for each species are provided. Ce – Cercopithecidae. B) Column1 – Common name (*Genus species*). Column2 – Genome assembly used. Column3 – Accession number for genome assembly. (PNG 1661 kb)

Additional file 7: **Supplemental file 2.** (pre)*GNA*- paralog presence before and after the 2R WGD in Vertebrata projected onto a Deuterostomia species tree. A) Sequence evidence of the six pre*GNA*- genes present in non-Vertebrata Deuterostomia; two Protostomia species, one Cnidaria, and one Placozoa species were included as outgroups (black and grey branches). These genes encode preGαi, o, q, v, s, and 12. The first number denotes the number of genes found. Small numbers denote the number of exons missing after curating the annotation as compared to the expected exon counts per phyla (specified at the top of the column). “/” separates multiple paralog gene copies (a, b, c, d). “,” indicate multiple transcripts variants exist which include different exons (.1 or .2), “~” indicate altered and/or erroneous exon borders as compared to other members within the same phylum. “?” indicate unclear paralog assignments due to missing exon data. B) Sequence evidence of individual paralogs after the radiation of Vertebrata. Only one species of pufferfish, turtle, and frog were interrogated if no ambiguity existed. Due to the debate of placement of the 2R WGD relative the emergence of Agnatha, it is not clear whether GNAI1–4, T1–4 and Q/11/14/15 are in fact 1:1 orthologs to Gnathostomata. Note: exonXL was not included in pre*GNAS* exon counts for a total of 12 exons, *GNAS* includes exonXL for 13 exons, *GNAS* in Placentalia possess 14 possible exons. *GNAL* possesses 13 exons for the alternatively spliced long and short exon1, pre*GNAV* possess 8 exons except in Cephalochordata while *GNAV* is encoded by 9 exons. *GNAZ* possess 2 exons. *pre*GNA12*, *GNA12, and *GNA13 exon counts vary across phyla, please refer to Fig. 6 for details. (PNG 1024 kb)

Additional file 8: **Supplemental file 3.** Transcriptome and Expression Data. All Deuterostomia gene sequences were validated by blasting against Expressed Sequence Tags (EST) and/or Transcriptome Shotgun Assembly (TSA) data when available [17, 21]. The tables show which species and paralogs were validated. The first number indicates the number of genes found per family (same as Supplemental file 2); the smaller characters represent EST/TSA data for each paralog. “@” indicates that a full-length or partial expression read fragment was found, “&” indicates a full-length or partial transcriptome read, “-” indicates no EST/TSA support was found. “/” separates multiple paralog gene copies (a, b, c, d) “,” indicate multiple transcript variants exist which include different exons (.1,.2), “*” indicates EST/TSA data did not include exon sequences for respective alternative transcripts (.1,.2). Dark blue/orange boxes indicate all paralogs were validated by partial or full EST/TSA hits, light blue/orange boxes indicate no reads were found to support that paralog. White boxes indicate that no EST or TSA data were available for analysis. Red boxes indicate EST and TSA data were found without sequence evidence for the gene present within the genome assembly. A) EST data. B) TSA data. (PNG 1711 kb)

Additional file 9: **Figure S7.** 5′ non-canonical splice site pattern of *GNAI1* intron6 in Sauropsida and Mammalia. A) Schematic representation of the primary transcript sequence of the *GNAI1* gene in Sauropsida and Mammalia with the start and stop codons as well as the SS explicitly shown. Possible untranslated regions (UTRs) are not shown. The representative exons (boxes) are drawn to approximate scale with their nucleotide length while introns (lines) are not drawn to scale. B) 5’ SS of intron6 in *GNAI1* of Sarcopterygii and spotted gar. The first seven nt of intron6 are highly conserved in all Mammalia and most Sauropsida (black box), while they vary in alligator, frogs and spotted gar (species marked in red). The intron sequence, and thus SS, is unknown for coelacanth. The first two nt of the boxed region constitute the SS pattern GC/GT. The figure was produced with the Jalview alignment viewer [30]. (PNG 2608 kb)

Additional file 10: **Figure S2.** Exon structure of *GNAI* and *GNAZ*. Most members of the Gαi family have a conserved gene structure with 8 protein-coding exons, similar exon lengths, and five conserved split codons shared across exons. The relative exon lengths of *GNAI* genes are represented by dark blue boxes. *GNAZ* only possesses two protein-coding exons (light blue). The first GNAZ exon sequence maps to exons 1–6 of *GNAI*, while the second *GNAZ* exon position maps to exons7 and 8 of *GNAI*. This exon-intron structure is indicative of a retrotransposition. The intron sequence may have been reinserted later into the gene to promote transcription. (PNG 152 kb)

Additional file 11: **Table S2.** Sites under positive selection in the branch leading to *GNAO.1*. Data is given for those residues that have a BEB probability for being in class 2a (sites under positive selection) for branch #1 (Fig. 9) > 90% in at least one of the tested codon models (F1X4, F3X4, Codon Table). The probabilities > 90% are marked in red. The identity and numbering of the residues in respect to the full-length protein sequence in human are given in column 1. (PNG 1046 kb)

Additional file 12: **Table S3.** Significant results of the branch-site model indicate positive selection in the *GNAO.1* #1 branch. The result of the likelihood ratio test was compared to a χ2 distribution with following significance levels * < 0.05, ** < 0.01, *** < 0.001 for each codon model tested (F1X4, F3X4, codon Table) in the #1 branch of *GNAO.1* (marked in Fig. 9). All other tested branches (#2, *1, *2, and *3) were not significant. Robustness of the parameter inferences (p0, p1, w0, wFG) was accessed by bootstrapping. BS = Branch-Site, LR = Likelihood Ratio, σ = standard deviation, Q_1_ = First Quantile (25th percentile), Q_2_ = Second Quantile (75th percentile). (PNG 52 kb)

Additional file 13: **Figure S3.** Sequence frequency logo of *GNAO* residues that were positively selected on the branch leading to *GNAO.1*. The duplication resulted in two pairs of exons7–8 that are mutually exclusive during splicing. Alternative splicing produces two transcript variants, *GNAO.1* and *GNAO.2*, that slightly differ in sequence. Some residues of the *GNAO.1* branch were positively selected after the duplication (branch #1 of Fig. 9). The identity of homologous positions is also shown for *GNAO* of Hemichordata, Echinodermata and Cephalochordata (lowest track). Teleostei and lampreys were excluded when testing for positive selection and when constructing the sequence logo. The sequence logo was created with Weblogo [123]. (PNG 824 kb)

Additional file 14: **Figure S4.** Exon 3 of *GNAS* in human. Expression of exon3 is supported by CCDS data. A region ~ 75 nt upstream and 25 nt downstream of the exon boundaries shows high levels of conservation in Placentalia. The same region is not conserved in non-placental Mammalia (platypus, wallaby and Tasmanian devil) as no BLASTz hits were retrieved (pink boxes). The Figure was created with the Ensembl webserver [15]. Bp - Basepair, CCDS - consensus coding sequence, GERP - Genomic Evolutionary Rate Profiling. (PNG 249 kb)

Additional file 15: **Table S4.** Retrogenes in Primates. The table summarizes the properties of *GNA*- retrogenes found in Primates. Two retrogenes (highlighted in bold) are the result of independent duplications of an existing retrogene. All other retrogenes are the result of a retrotransposition event. The retrogene name, location, location of the parent and the proximity to a promotor are given for human unless specified differently in parenthesis. The retrogene is situated next to the gene specified in the synteny column for the phylogenetic group given in the column ‘LCA’ (last common ancestor). Requiring conservation within the complete phylogenetic group, the coding potential of the respective region was evaluated with RNAcode 40] (+: methionine contained in open reading frame, ORF; −: no methionine in ORF). Conserved ORFs that are similar to the parent ORF were detected via *blastn* with the human parent gene as query. Expression was accessed by interrogating the Expression atlas database restricting to RPKM > 0.5 and additionally other sources for non-human Primates. Given is the number of experiments, the number of conditions (in parenthesis) and the number of tissues (last value) in the last two columns. Cja – *Callithrix jacchus*, Ggo – *Gorilla gorilla*, Csy – *Tarsius syrichta*, Mmu – *Macaca mulatta*, Pan – *Pongo abelii,* Csa – *Chorocebus sabaeus*. (PNG 1280 kb)

Additional file 16: **Figure S5.** Expression level heatmap of *GNA*-retrogenes and parent genes in different Primates. We depict a selection of RNA-Seq datasets which show expression of the respective *GNA*- retrogenes with RPKM > 0.5. The color scheme depicts orthology relationships. Note that the dark brown paralog is the results of an independent duplication of *GS1-124 K5.9*. A) RNA-Seq experiment of 16 human individual tissues and mixture from the Illumina Body Map (primarily Caucasian origins from both sexes, ages 19–86) [124]. B) RNA-Seq experiment of 9 rhesus macaque tissues from Merkin et al. (male, unknown age) [125]. C) RNA-Seq experiment of 13 human tissues from the ENCODE project (both sexes, 21–66 years) [126]. D) RNA-Seq of 14 tissues of olive baboon from the non-human Primates reference transcriptome resource project (female, 6 years) [127]. E) RNA-Seq experiment in 14 human brain tissues from the Human Developmental Biology Resource (both sexes, 10 weeks post conception) [128], F) RNA-Seq experiment of 5 vervet-AGM tissues (male, 3 years). (PNG 1640 kb)

Additional file 17: **Figure S6.** Local exon duplications of *GNAQ*, *GNA11*, and *preGNAI*. A) Alternative splicing of two mutually exclusive exon4 of *GNAQ* and *GNA11* results in two different RNA transcripts represented. Box lengths correlate with average curated exon lengths (intron line lengths do not correspond to intron lengths). B) Tertiary crystal structure of Mammalia Gαq (taupe) with exon4 (orange) borders mapped with RGS protein interaction removed (top) and with RGS present (bottom - ruby) (PDBID 5D09 [54]). Alternatively, spliced exon4 provides sequence diversity for critical protein-protein interfaces such as the RGS protein (purple). C) ML trees of nucleotide sequences from exon4 of *GNAQ*/*GNA11and* exon5 of *GNAI* across basal Chordata. (PNG 1933 kb)

Additional file 18: **Figure S8.** DNA- and RNA-binding protein motifs overlapping with the 5′ non-canonical splice site of intron6 in *GNAI1*. A) Local enrichment of known DNA-binding protein (DBP) motifs in comparison to a uniform motif distribution are shown for Sarcopterygii with ‘GC’ SS (positive set) versus lobe-finned fish and spotted gar with ‘GT’ splice site (SS) (control, adjusted *p*-value < 0.05). The shown motifs are either present in all species of the positive set and in none of the controls (PRDM1_full, FXR1) or follow this rule with at most one exception. Mafk_secondary UP0004_2 (red), NFIX_full_3 (dark blue), PRDM1_full (green), STAT2:STAT1 (pink, behind green). B) Local enrichment of known RNA-binding protein (RBP) motifs in comparison to a uniform motif distribution. FXR1 (lime green). The SS is located at position 45 along the x-axis. Sequence positions < 45 correspond to exon6, while positions > 45 correspond to intron6. The y-axis indicates the probability of a DBP/RBP motif present centrally at the indicated position for the positive set (solid line) and the control set (dotted line). None of the motifs occurs surprisingly more often at a specific position in the positive set than in the control set (Fisher’s exact test, adjusted p-value < 0.05). The Figure was created with Centrimo [36]. (PNG 287 kb)

Additional file 19: **Figure S9.** Implications of alternative exon usage on tertiary structure in Cephalochordata preGαi and preGαq. A) Mutually exclusive inclusion of Cephalochordata exon6.1 and 6.2 in *preGNAI* (blue) yields two different transcripts during alternative splicing. Representative box lengths correlate with the average curated exon lengths (intron lines do not). B) Mutually exclusive inclusion of Cephalochordata exon5.1 and 5.2 in *preGNAQ* (beige) also yields two different transcripts during alternative splicing. C) Splice variant exon borders mapped onto two Gαi crystal structures (PDB IDs 1GP2 [56] and 1AGR [57], respectively). The sequence encoded by exon6 (light blue) influences the interface between the Gβγ subunits of the heterotrimer (crimson/grey - left) and downstream effector protein partners such as the RGS protein (purple – right). D) Splice variant exon borders mapped onto two Gαq crystal structures. The sequence encoded by exon5 (orange) influences the protein interfaces between effector proteins such as PLC (lavender – left) and RGS (purple - right) (PDB IDs 4QJ3 [53] and 5DO9 [54]). E) ML tree of (*pre)GNAI*/*GNAQ* exons indicates both duplications were independent. (PNG 1799 kb)

Additional file 20: **Figure S10.** Implications of alternative exon usage on tertiary structure in Cephalochordata preGαs. A) Alternative splicing of Cephalochordata exon12 and 13 in *preGNAS* (green) yields two different mutually exclusive transcripts. B) Splice variant exon borders (dark green) mapped onto a Gαs structural model bound to the G protein βγ subunits (crimson/grey) and a GPCR (pink) respectively and rotated 90°. (PNG 2085 kb)

Additional file 21: **Figure S11.** DNA- and RNA-binding protein motifs overlapping with the 3` canonical and non-canonical splice sites of intron 3 in *GNAS*. All included motifs are predicted to occur in the positive set (for six Placentalia), but not at the same position in the control set (eight non-Placentalia Sarcopterygii). Note, that some motifs occur in the control set, but at a different position than in the positive set, e.g. Gata4. The shown motifs overlap with the conserved intronic region upstream of exon 4. A) Local enrichment of known DNA-binding protein motifs (DBP) in comparison to a uniform distribution of motifs (E-value < 1, adjusted p-value < 0.05). Gata4 (blue), Mybl1_secondary (pink), GATA3_full (red), Sox4_secondary (green), FOXP1 (turquoise). B) Local enrichment of known RNA-binding protein motifs in comparison to a uniform distribution of motifs (E-value < 1, adjusted p-value < 0.05). PCBP1 (light blue), U2AF2 (light green), RBM47 (dark green). The non-canonical splice site is located at position − 7. Sequence positions < − 7 belong to intron 2 while positions > − 7 belong to exon 4. The y-axis indicates the probability of a DBP/RBP binding centrally at the indicated position for the positive set (solid lines) and for the control (dotted lines). None of the motifs occurs more often at a specific position in the positive set than in the control set (Fisher’s exact test, adjusted p-value < 0.05). The Figure was created with Centrimo [38]. (PNG 410 kb)

Additional file 22: **Figure S12.** DNA- and RNA-binding protein motifs overlapping with the extended conserved region around exon 3 in *GNAS* of 33 Placentalia. Exon 3 is located at positions 0–46 on the x-axis. A) Local enrichment of known DNA-binding protein (DBP) motifs in comparison to a uniform motif distribution. 30 motifs are enriched in the reported region with a E-value < 0.0001 in all investigated Placentalia; only a subset of these is shown for clarity: Gfi1 (light blue), Hltf (dark blue), EGR1 (pink), MZF1_5–13 (light green), En1 (red), E2F4 (orange), Hoxc9 (dark green). B) Local enrichment of known RNA-binding protein (RBP) motifs in comparison to a uniform motif distribution. Nine motifs are enriched in the reported region with an E-value < 0.0001 in all investigated Placentalia. TARDBP (light blue), DAZAP1 (dark blue), PPRC1 (pink), SRSF9 (light green), SRSF10 (red), CNOT4 (orange), PCBP1 (dark green), KHDRBS1 (black), RBM38 (purple). Note that the SRSF9 binding site is located within the exon and does not overlap with either splice site. C) Local enrichment of RBP sites predicted by Pollard et al. [112]. The respective motifs do not occur in all investigated Placentalia as indicated by a lower probability. SRSF2 (dark blue), SRSF1 (light blue), HNRNPA1 (pink). The 3′ ‘AG’ SS is located at position 0 along the x-axis. The y-axis indicates the probability of a DBP/RBP motif being located centrally at this position. The Figure was created with Centrimo [38]. (PNG 598 kb)

## Appendix A

### i – Lineage-specific duplications across Metazoa shed light on gene flexibility and duplication integrity

Multiple duplication events occurred in *C. elegans* resulting in over 20 copies of *GNA*- like genes (named *GPA*- in *C. elegans*). We included previously annotated *GPA*s. However, only four genes appear to be similar to the five primary Gα families of Vertebrata; the rest cluster into two separate branches on the ML tree (black subtrees Fig. 2, Additional file 2: Supplemental file 1). *GPA-4* and *GPA-16* are sequentially similar to *preGNAI*, though their exon border positions differ from the conserved eight protein-coding exons found within this family. They nest within the *preGNAI* branch with bootstrap values (BS) of 55. *GOA* and *GPA-12* may be orthologs of *preGNAO* and *preGNA12*, respectively, despite both genes possessing altered exon positions relative to the other non-Deuterostomia genes included; both possess moderate BS values (85 and 86) and form separate monophyletic groups with other *preGNAO* and *preGNA12* members, respectively.

Of note, there are other lineage-specific tandem duplications found for *preGNAI* within Placozoa (*T. adhaerens*). We found evidence of *preGNAI* tandemly duplicating into three copies (copy *a* and *b* are side by side, and the third ‘*c’* copy lies ~ 116,000 nt downstream). All three copies of *preGNAI* maintain the same exon-intron structure (eight protein-coding exons with five split codons). As mentioned before, the *preGNAIa* and *b* copies group within the *preGNAI* subtree as an independent branch, while copy ‘*c*’ forms the base of the *GNAZ* tree. Despite the location of *preGNAIc* on the ML tree, it is unlikely that it is a progenitor to the Vertebrata *GNAZ* due to its absence in all other non-Vertebrata Metazoa lineages investigated. In addition, *GNAZ* genes are situated within the intron of *RSPH14* genes in Vertebrata. Introns of *RSPH14*-like sequences found in all non-Vertebrata Deuterostomia branches did not possess traces of this *preGNAIc* gene or any other *preGNAZ*-like gene. Taken together this suggests that *preGNAIc* is the result of an independent, local gene duplication event which occurred within *T. adhaerens*. Therefore, we term the third copy of *preGNAI* in Placozoa as *preGNAIc* and not *preGNAZ*.

The putative fourth copy of *preGNAI* in *T. adhaerens* was identified as *preGNAO* which lies on a different scaffold roughly 750,000 nt upstream of *preGNAS*. *preGNA12* is also tandemly duplicated into two adjacent genes. These gene copies are arranged in a head to tail orientation on the same scaffold roughly 3000 nt apart.

*preGNAI* duplications in Cnidaria, *N. vectensis*, are not tandem, but rather are located on separate scaffolds of either 46,000 or 37,000 nt in size. The multiple gene copies all appear to be lineage-specific duplications, as the ML tree shows both *a* and *b* copies forming their own separate branch, independent of the *T. adhaerens* tandemly duplicated genes. As they all maintain the conserved exon-intron structures specific to *GNAI*, these data support our hypothesis of *preGNAI* and *preGNAQ* differentiating into separate genes before the emergence of Holozoa.

Only one possible duplication event of *preGNAQ* was found within the investigated non-Deuterostomia species. A gene fragment in *B. schlosseri* was found (missing 4.5 out of 7 exons) which groups within the *GNA15* subtree (Gαq family). Due to the lack of available synteny, transcriptome and expression data, it is inconclusive whether this gene is a pseudogene or a full-length *preGNA*- gene.

Two copies of *preGNAS* genes were identified within *D. melanogaster* (denoted *GalphaS* and *GalphaF*). These genes do not share synteny and are located on chromosomes 2R and 3 L, respectively. In addition, *GalphaS* only shares seven exon border positions with the gene structure found for *preGNAS*; *GalphaF* maintains only three.

Two copies of *preGNAS* were found in both species of Echinodermata investigated and in Hemichordata suggesting a local duplication of this gene occurred within non-Chordata Deuterostomia which maintained its exon-intron structure. We created a ML tree of all sequences found through the EMS pipeline (Fig. 2). We find that these *preGNAS* are situated at the root of the *GNAS*/*GNAL* branch of Vertebrata. Nevertheless, the *preGNAS* do not form a monophyletic group. In addition, none of *preGNAS* duplications appear to share synteny, though lack of large contig size for all paralogs prevents a thorough analysis of gene neighbors.

The duplication of *preGNA12*, found in *P. miniata* (bat star starfish), also does not appear to be arranged in tandem. It does not appear to be a progenitor to the Vertebrata *GNA13* as it groups tightly to *preGNA12* genes found in Echinodermata and other non-Vertebrata *preGNA12* genes.

Two *preGNAV*-like sequence fragments were found within *S. kowalevskii* and *B. belcheri* (Hemichordata and Cephalochordata, respectively). The sequence fragments maintain some conserved exon border positions indicative of *preGNAV*. However, the small contig size and missing data prevented identification of start codons within the sequences. Therefore, it is unclear if these fragments are true protein-coding genes which were not fully assembled or they represent pseudogene remnants of a parent *preGNAV* duplication. Within the ML tree, these two gene fragments are situated between the (*pre*) *GNAV* and (*pre*) *GNAS* subtrees.

In Urochordata, we find evidence of multiple independent duplications which led to the reinsertion of *preGNAI* into different regions of the genome as an intronless gene or as fragments which maintained some exon border positions. In two species (*C. intestinalis* and *C. savignyi*), several different reinsertions were found that group within the *preGNAI* branch. Synteny mapping was unsuccessful in distinguishing gene neighbors around these two paralogs. In *B. schlosseri* other gene duplications and fragments were found; these genes are sequentially distinct from those found in the *C. intestinalis* and *C. savignyi* species. Two nest within the *preGNAI* branch, one appears to be a fragment of a *preGNAV*-like gene, while the fourth gene, though it maintains some *preGNAI*/*GNAV*-like exon border positions groups between the *GNAV* and *GNAS* subtrees.

### ii – Individual, local exon duplication across *preGNA*- paralogs in Cephalochordata

In addition to the local, full-length duplications of (*pre*)*GNA*- genes in different branches, we also found evidence of smaller duplications involving individual exons within some of the (*pre*)*GNA*- genes. These exon duplications gave rise to alternative transcripts with different mutually exclusive exons.

In *preGNAI*, we found evidence of exon6 being duplicated, while in *preGNAQ* exon5 was duplicated in both species of Cephalochordata (*B. floridae* and *B. belcheri*) (Additional file 19: Figure S9a-b). Though exon6 of *preGNAI* and exon5 of *preGNAQ* correspond to homologous sequence regions, we did not find evidence of this exon duplication arising before the emergence of separate *preGNAI* and *preGNAQ* genes (pre-Metazoa divergence). Instead it appears that both alternative splicing events arose independently at the base of the Cephalochordata lineage. All tests for recombination/gene conversion were negative.

If this exon duplication had occurred before the divergence of *preGNAI* and *preGNAQ* (within *preGNAI/Q*), all other non-Cephalochordata Metazoa lineages would have each independently lost one of the duplicated exons, exon6 in *preGNAI* and exon5 in *preGNAQ*.

To test this unlikely scenario, we built ML trees of the non-Vertebrata Deuterostomia *preGNAI* and *preGNAQ* nucleotide sequences which corresponded to the mutually exclusive exons, exon6 or exon5, respectively. If the duplication of this exon occurred in *preGNAI/Q*, we expect the mutually exclusive exons (.1 and .2) of *preGNAI* and *preGNAQ* to be more akin to each other across species than to their own family, (*pre*) *GNAI* or (*pre*) *GNAQ*, respectively. Instead, we see that each of the mutually exclusive exons is more closely related to the other members of its own family (Additional file 19: Figure S9e, Additional file 5: Supplemental file 6). This suggests that exon 5 of *preGNAI* was independently duplicated of the *preGNAQ* duplication of exon6, and both occurred within the Cephalochordata lineage.

We then compared the number of per site nucleotide substitutions that arose since the split of *preGNAI* and *preGNAQ* until the speciation of both Cephalochordata to create two sets of sequences: exons 5/6 vs. all other exons. The average nucleotide substitution rate of exons 5/6 is roughly equal to the substitution rate for all other exons (0.6 vs. 0.57). In contrast, the average rate of nucleotide substitution is higher for the branches leading to *preGNAQ* exon5.1 and *preGNAI* exon6.1, respectively, than for the other exons (0.69, 0.65). This suggests an increased substitution rate in the branches leading to *preGNAQ* exon5.1 and *preGNAI* exon6.1 after the exon duplication.

These exons correspond to protein sequences in critical regions of the tertiary fold necessary for protein-protein interactions. Such interfaces are necessary for binding the Gβγ subunits, Regulators of G protein signaling (RGS), Phospholipase C (PLC) and other downstream effector proteins (Additional file 19: Figure S9c-d). Therefore, these two independent exon duplication events may have allowed for the evolution of new functionality by increasing sequence diversity within Cephalochordata.

The Cephalochordata lineage appears to have undergone several such local exon duplication events. We found evidence of alternative splicing of the final two exons of *preGNAS* in both species of Cephalochordata investigated (Additional file 20: Figure S10). The second of the exon pairs (12.2 and 13.2) encodes additional three nucleotides resulting in the extension of Gαs by one amino acid within the C-terminus. Though the sequences have diverged, they still maintain several highly conserved motifs and high sequence similarity (80–81% at the amino acid level, 88–89% at the nucleotide level).

These exons encode sequences of the α5 helix, which is important for GPCR interaction and specificity [86, 111]. The ability to alternatively encode two different C-terminal exons for these transcripts may impact the diversity of receptors with which the preGαs subunit may interact. In addition, movement of the secondary structural element, the α5 helix, has been shown to be necessary for subsequent G protein activation after coupling with the receptor [58, 101]. The resulting different protein isoforms may therefore have different abilities to bind GPCRs, respond to and undergo the necessary conformational changes to activate as their α5 and α4 helices and β6 strand differ in sequence. Additional file 20: Figure S10b shows these exon borders mapped to available tertiary structural models of a Gαs protein. These two exons (dark green) overlay with regions necessary for receptor interaction and subsequent G protein activation necessary for signal propagation.

## Appendix B

### i – The intron of *GNAZ* does not show traces of a transposon insertion

In order to clarify the origin of *GNAZ*’s intron, we checked whether this intron could have originated from the insertion of a transposon. This mechanism often leaves traces within the exonic sequence in the form of a conserved ‘AG’ as last nucleotides of the upstream exon and ‘GT’ as first nucleotides of the downstream exon [112, 113]. The transposon preferentially cuts downstream of the ‘AGGT’ consensus sequence and then inserts into this genomic position. Two of the nucleotides of the consensus sequence then become part of the intron on either side of the transposon sequence resulting in the following intron sequence: ‘GT-transposon-AG’. We evaluated the conservation of these residues in all *GNAZ*; however, none were found to be conserved. Several alternative mechanisms for gaining introns exist, e.g. intron transposition and intronization. These alternative possibilities were not evaluated due to the sparse species sampling (and thus high divergence) within this position of the tree. Therefore, the origin of the exon-intron structure of *GNAZ* remains an open question.

### ii – Conservation of nucleotides flanking exon3 and the 5′ end of exon4 in *GNAS* overlap with DNA-/RNA-binding protein motifs

Interestingly, not only the sequence of exon3 of *GNAS*, but also the surrounding intronic sequences (3′ 75 nt of intron2 and 5′ 25 nt of intron3) are conserved in Placentalia (Fig. 10, Additional file 14: Figure S4). A similar pattern of conservation is observed for the 3′ 20 nt of intron3 adjacent to exon4 in Placentalia. In contrast, there are no conserved regions within the 5′ end of intron2. The conserved genomic footprints suggested external pressures were constraining the nucleotides surrounding exons3 and 4.

We tested for local enrichment of DBP and RBP motifs at these three SS including the conserved nucleotides of the introns. Near the 3’ SS of intron3, five DBP motifs as well as three RBP motifs are locally enriched within the intronic, conserved nucleotide region or overlapping with the SS in Placentalia in comparison to a uniform background distribution (adjusted *p*-value < 0.05, Additional file 21: Figure S11). The binding sites of the transcription factors Gata3 and 4, which are involved in myogenesis [114] partially overlap with the non-canonical SS.

The recognition of a 3′ ‘TG’ SS by the U2 spliceosome is highly unusual (0.016% of U2 SS) [115], but well documented for *GNAS*. A previous study showed that the usage of the ‘TG’ SS is promoted by the splicing factor SF2/ASF that has been suggested to bind within exon3 of *GNAS* [116]. SF2/ASF has an antagonistic relationship with another splicing factor, hnRNPA1, which is also suggested to bind in exon3. Our current study confirms this functional connection of exon3 and the ‘TG’ SS by phylogenetic co-occurrence. Investigation of RBP and DBP sites within exon3 and the surrounding, conserved intronic sequence suggest the conservation of the SF2/ASF binding site (SRSF1) within exon3 in 31 out of 33 species of Placentalia and conservation of the hnRNPA1 binding site in 32 Placentalia (Additional file 22: Figure S12c). The hnRNPA1 binding site is situated in the conserved intronic region upstream of exon3. The 3’ SS region of intron2 and the 5’ SS region of intron3 harbor 30 DBP motifs and nine RBP motifs that are locally enriched in the reported region in all 33 investigated species of Placentalia (adjusted *p*-value < 0.05, Additional file 22: Figure S12a-b). We additionally tested for motif enrichment in the whole region with AME encompassing exon2, intron2, exon3, intron3 (when available) and exon4 in Placentalia in comparison to non-Placentalia Sarcopterygii. No DBP or RBP motifs were significantly enriched (adjusted *p*-value < 0.05).

The conservation of 100–300 intronic nucleotides surrounding cassette exons has been observed previously and used as a predictor for alternative splicing levels leading to the inclusion or exclusion of the respective exons in several large-scale studies [117, 118]. Nevertheless, Wainberg et al. noticed that there is only little overlap of over-represented 6-mers from these conserved, intronic regions with known RBP motifs [118]. A full mechanistic explanation of the observed conservation pattern is the focus of current research.

### iii – Local exon duplications add sequence variety and potential functional divergence for *GNAQ*, *GNA11* and *preGNAI*

We found exon duplications in exon4 of *GNAQ* in coelacanth and gar as well as *GNA11* in coelacanth, gar, and Teleostei (see below for exceptions) (Additional file 17: Figure S6a). The duplicated exons allow for the inclusion of either exon4.1 or exon4.2 during alternative splicing. As this exon duplication is present in coelacanth and gar for both paralogs, we propose that this duplication occurred before the 2R WGD in *preGNAQ*, but was subsequently deleted in the other Vertebrata lineages of *GNAQ* and *GNA11* (e.g. within the Agnatha lineage). Upon 3R WGD, at the base of Teleostei, *GNAQ* lost one variant of exon4; therefore, no Teleostei *GNAQ* exon4 duplications exist. However, both *GNA11* exon variants were retained in one gene copy of zebrafish and cod (*GNA11a.1*,*.2* and *GNA11b.1*) and in both gene copies of medaka, stickleback and pufferfish (*GNA11a.1*,*.2* and *GNA11b.1*,*.2*).

Surprisingly, the homologous sequence of *preGNAI*, encoded by exon5, can also be alternatively spliced in all four investigated Urochordata species. To test whether those exon duplications originated from one, two or three independent duplication events, we constructed ML trees of nucleotide sequences from exon5 of (*pre*) *GNAI* and exon4 of (*pre*) *GNAQ* and *GNA11* from Deuterostomia (excluding Tetrapoda sequences, Additional file 17: Figure S6c). As discussed with the local exon duplications found in the Cephalochordata lineage, it is expected that if just one single exon duplication occurred before the gene duplication and divergence of *preGNAI/Q* into *preGNAI* and *preGNAQ*, the two exon variants, .1 and .2, would be more similar within their exon variant group than to their subfamily counterparts. Instead, *preGNAI* variants are independent nodes outside of the Gαq family and are not nested within any other branch supporting two independent, local duplication events. We find that the ancestral branch of *preGNAQ* orthologs from Urochordata, Cephalochordata, and Hemichordata bifurcates into two main branches composed of *GNAQ* and *GNA11*; one subtree branches into the .1 variant while the other branches to become the .2 variant cluster. This tree topology supports a scenario, where the second local exon duplication occurred before the 2R WGD which resulted in *GNAQ* and *GNA11*.

This indicates an especially high susceptibility for this region to be retained after local exon duplication. The protein segment encoded by these exon sequences mediates the interaction of Gα with the Gβγ subunits and multiple downstream-signaling effector proteins such as the RGS or PLC proteins as shown from the overlay of these exon positions onto the tertiary protein structures (Additional file 17: Figure S6b). Such interaction is necessary for G protein heterotrimer formation [56, 119], interaction with the GPCR [119, 120], and ultimately signal cessation and complex reformation [120]. The ability to alternatively splice this region, and increase the sequence diversity of the Gαq and Gα11 proteins could alter which Gβγ subunits bind or which downstream signaling cascades are initiated by these Gα subunits.

### iv – Conserved Non-canonical Splice Sites contain putative motifs for DNA-/RNA-binding proteins

Flanking most exons are highly conserved SS sequence patterns which direct the binding of the splicing machinery and thus mediate the removal of introns out of the primary RNA transcripts [121]. The canonical SS ‘GT’ is found immediately downstream of the transcribed exon (5’ SS of the intron) while ‘AG’ is found upstream of an exon (3′ intron SS) in 98.93% of all splicing events in Vertebrata [115]. We found conservation of these canonical ‘GT-AG’ splicing patterns for all of the exon sequences annotated with two exceptions. The first is the alternative upstream SS of exon4 in *GNAS* in Placentalia which has been discussed above.

In addition, we found the highly conserved 5′ non-canonical SS ‘GC’ in intron6 of *GNAI1* in most species of Sauropsida and Mammalia (Additional file 9: Figure S7). ‘GC-AG’ represents 0.89% of all splicing events, making it the most common SS in Vertebrata after ‘GT-AG’ [115]. This non-canonical SS is present in neither species of Amphibia investigated (*X. tropicalis* and *X. laevis*) nor in alligator. All Actinopterygii investigated possess the canonical ‘GT’ 5’ SS for intron6; however, this region is unresolved in the coelacanth genome preventing dating of exact origin of this non-canonical SS.

The emergence of the non-canonical SS in *GNAI1* co-occurs with the conservation of the extended ‘GC’ SS consensus motif: ‘A**A**G’ (exonic) and ‘GCAAGT’ (intronic) with one substitution in the exonic region indicated in bold [122]. These nucleotides are not conserved in Deuterostomia possessing the canonical SS (Additional file 9: Figure S7). It can be excluded that the non-canonical SS is involved in the skipping of exon6 as no such isoform is supported by EST or TSA data. The conservation of the extended ‘GC-AG’ SS consensus motif thus promotes splicing of exon6, and it is not involved in alternative splicing.

The observed switch from a canonical to a non-canonical SS and its systematic conservation is surprising. Therefore, we evaluated potential selective pressures acting on nucleotides surrounding the SS, e.g. to maintain binding sites of RBPs or DBPs requiring its strict conservation in Mammalia and Sauropsida. Non-canonical SS may also regulate tissue-specific expression and alternative splicing efficiency [115]; *GNAI1* has widely distributed Mammalia tissue expression [75] and no functional alternative transcripts were found for this gene.

To evaluate other potential selection pressures present in this region, we compared the nucleotide sequences surrounding the non-canonical SS to species possessing the canonical SS and scanned for local enrichment of DBP and RBP motifs. We uncovered four potential transcription factor binding sites (Additional file 18: Figure S8a) and one RBP motif (Additional file 18: Figure S8b) that overlap with the respective non-canonical SS region. These binding motifs are strictly conserved in all Mammalia and Sauropsida genomes (except for alligator) yet are not conserved in the four control species with canonical SS. The only exception being the RBP motif for FXR1; this motif seems to be shifted, making binding as equally likely in comparison to the control. The other DBPs all have a reduced binding probability in the control species. Although these motifs show an interesting distribution across the positive and control set, none of the binding motifs are seen more often in the positive than in the control set (Fisher’s exact test, adjusted-value < 0.05). Therefore, experimental validation is necessary to infer the roles of these *cis*-regulatory factors in transcription and splicing of *GNAI1*.

## Abbreviations

2R WGD: 2nd (and 1st) Round of Whole Genome Duplication in the Vertebrata ancestor
3R WGD: 3rd Round of Whole Genome Duplication in the Teleostei ancestor
AA: Amino Acid
BS: Bootstrap
DBP: DNA binding protein
EMS: ExonMatchSolver
EST: Expressed Sequence Tags
GPCR: G protein Coupled Receptor
ML: Maximum Likelihood
nt: Nucleotide
ORF: Open reading frame
PLCβ: Phospholipase Cβ
RBP: RNA binding protein
RGS: Regulator of G protein Signaling
SS: Splice Site
TCE: Translated Coding Exon
TSA: Transcriptome Shotgun Assembly
XL: Extra-long exon1 (*GNAS* and *GNAL*)
XXL: Extra-extra-long exon1 (*GNAS*)
