# Supplementary figures and images for: Tracing the evolution of the heterotrimeric G protein α subunit in Metazoa
Source: BMC Evol Biol. 2018 Apr 11;18:51. doi: 10.1186/s12862-018-1147-8 (PMC5896119; doi:10.1186/s12862-018-1147-8)

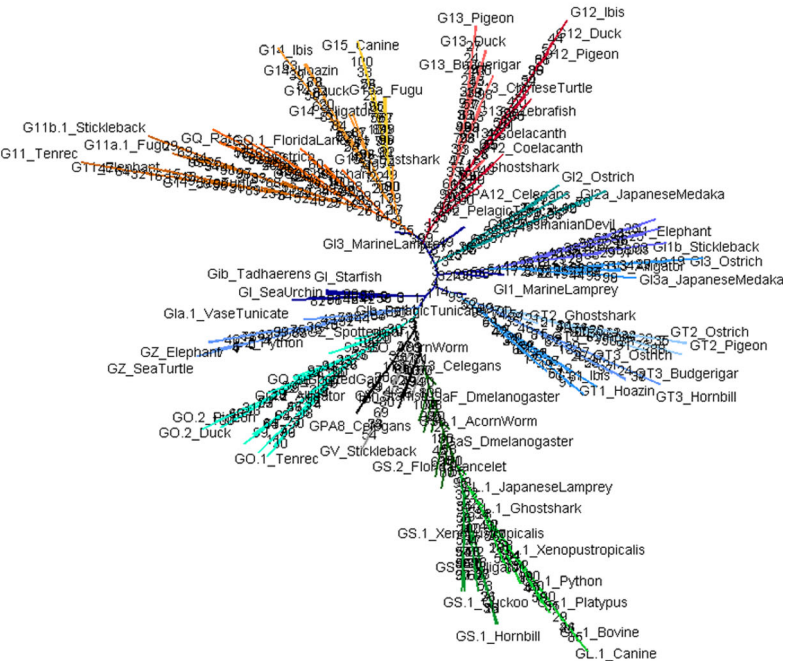

Supplement: Supplementary file 2 — Supplemental file 1. Maximum Likelihood Tree of (pre)GNA- genes. ML tree built with all paralogs and sequences evaluated in Nexml format. Bootstrapped replicates were summarized into Extended Majority Rule Consensus Trees and reported with bootstrap (BS) values. (PDF 4751 kb) [file 12862_2018_1147_MOESM2_ESM.pdf]

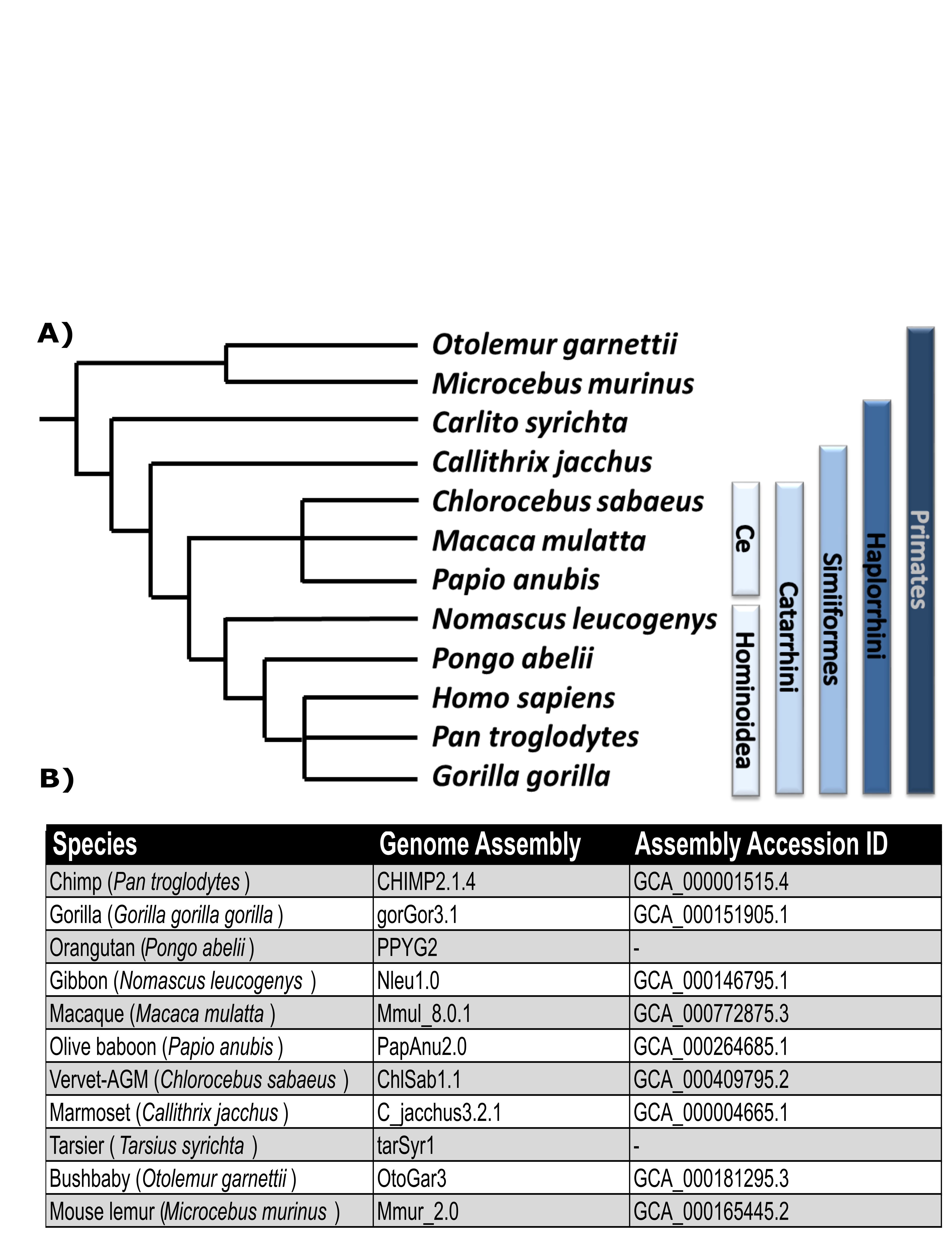

Supplement: Supplementary file 6 — Figure S1. Primates species investigated for retrogenes. The existence of GNA- pseudogenes was investigated within human and 11 other Primates species. A) Primates species investigated. The Latin names and clades for each species are provided. Ce – Cercopithecidae. B) Column1 – Common name (Genus species). Column2 – Genome assembly used. Column3 – Accession number for genome assembly. (PNG 1661 kb) [file 12862_2018_1147_MOESM6_ESM.png]

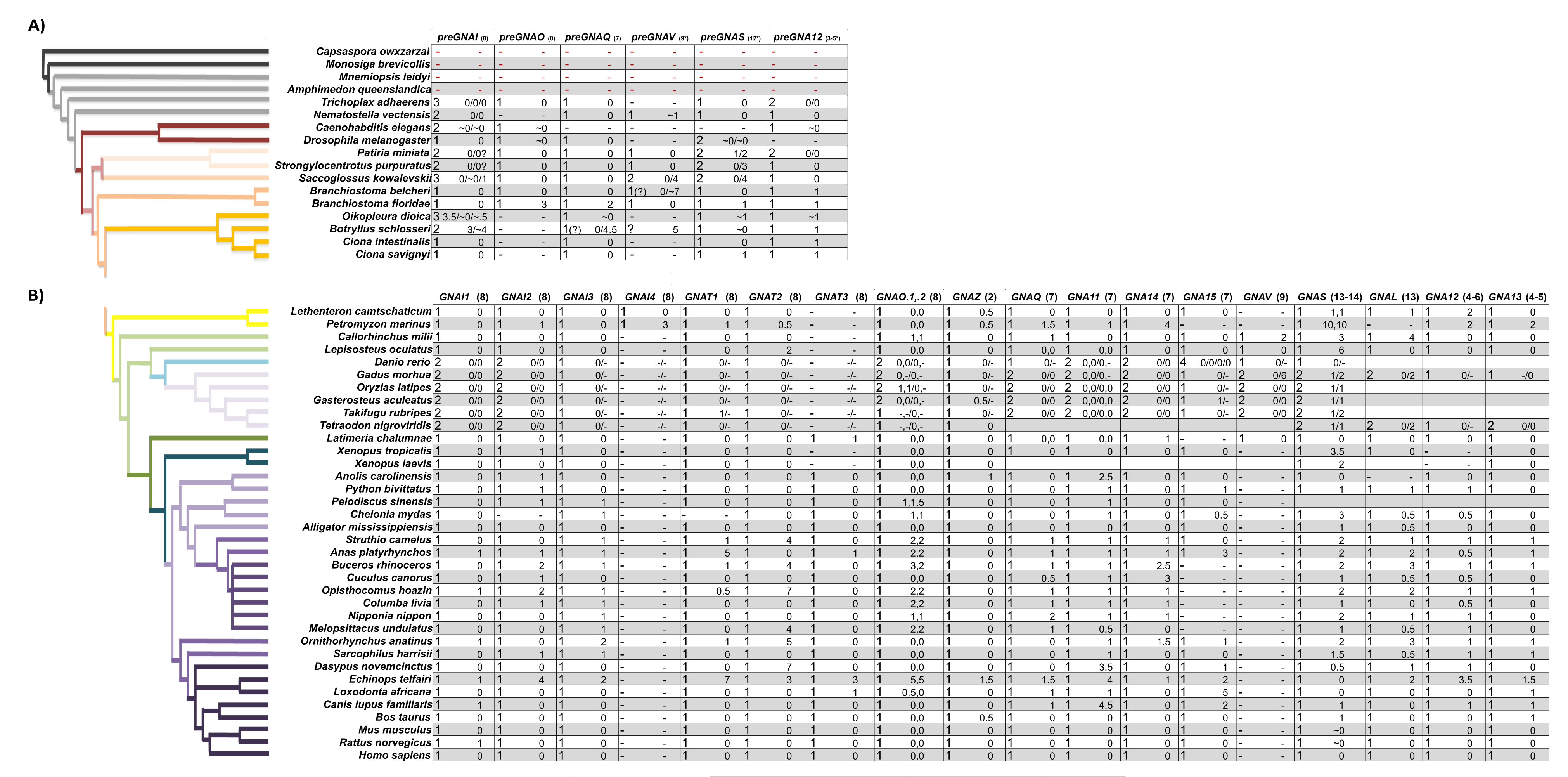

Supplement: Supplementary file 7 — Supplemental file 2. (pre)GNA- paralog presence before and after the 2R WGD in Vertebrata projected onto a Deuterostomia species tree. A) Sequence evidence of the six preGNA- genes present in non-Vertebrata Deuterostomia; two Protostomia species, one Cnidaria, and one Placozoa species were included as outgroups (black and grey branches). These genes encode preGαi, o, q, v, s, and 12. The first number denotes the number of genes found. Small numbers denote the number of exons missing after curating the annotation as compared to the expected exon counts per phyla (specified at the top of the column). “/” separates multiple paralog gene copies (a, b, c, d). “,” indicate multiple transcripts variants exist which include different exons (.1 or .2), “~” indicate altered and/or erroneous exon borders as compared to other members within the same phylum. “?” indicate unclear paralog assignments due to missing exon data. B) Sequence evidence of individual paralogs after the radiation of Vertebrata. Only one species of pufferfish, turtle, and frog were interrogated if no ambiguity existed. Due to the debate of placement of the 2R WGD relative the emergence of Agnatha, it is not clear whether GNAI1–4, T1–4 and Q/11/14/15 are in fact 1:1 orthologs to Gnathostomata. Note: exonXL was not included in preGNAS exon counts for a total of 12 exons, GNAS includes exonXL for 13 exons, GNAS in Placentalia possess 14 possible exons. GNAL possesses 13 exons for the alternatively spliced long and short exon1, preGNAV possess 8 exons except in Cephalochordata while GNAV is encoded by 9 exons. GNAZ possess 2 exons. *preGNA12, *GNA12, and *GNA13 exon counts vary across phyla, please refer to Fig. 6 for details. (PNG 1024 kb) [file 12862_2018_1147_MOESM7_ESM.png]

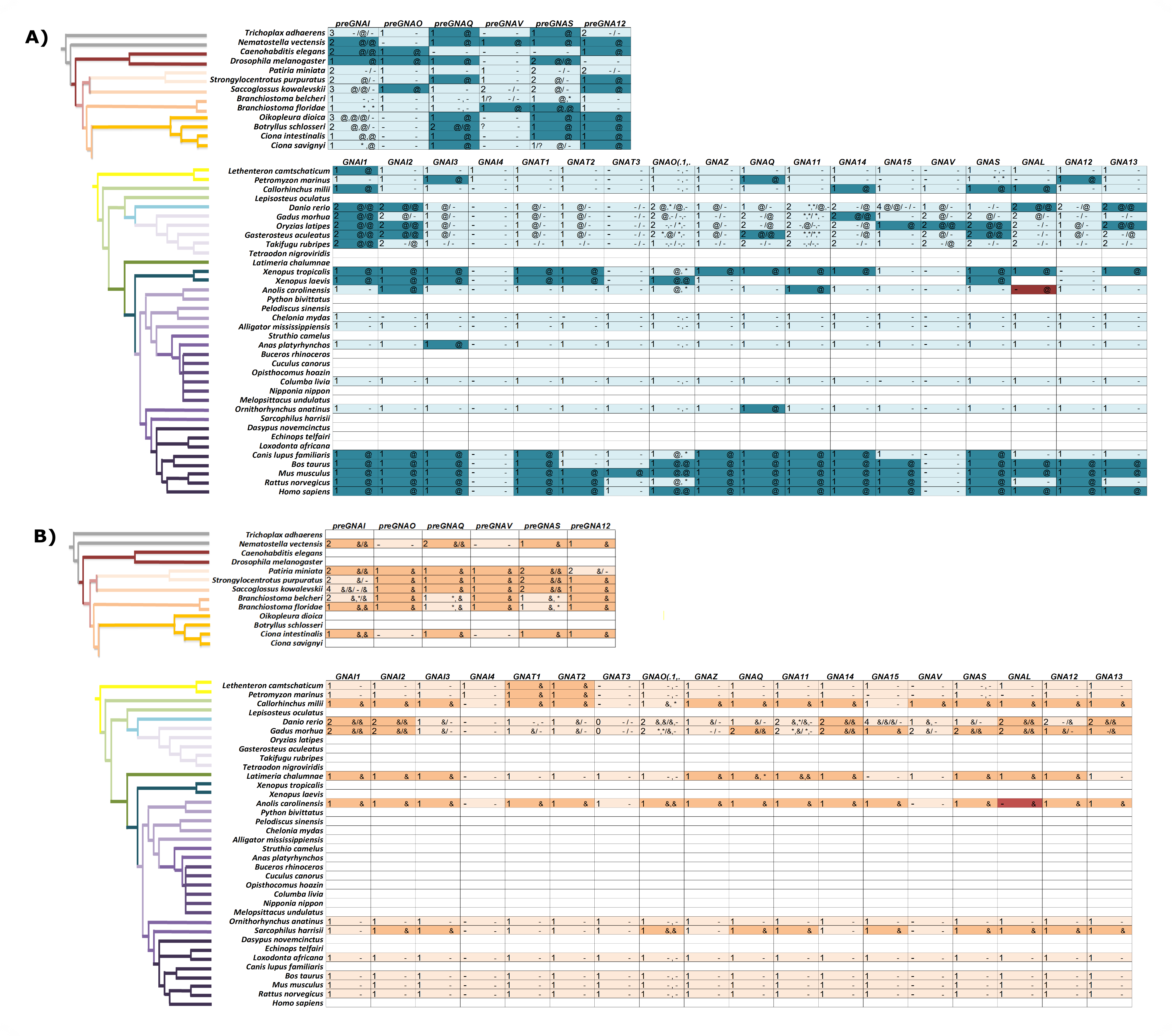

Supplement: Supplementary file 8 — Supplemental file 3. Transcriptome and Expression Data. All Deuterostomia gene sequences were validated by blasting against Expressed Sequence Tags (EST) and/or Transcriptome Shotgun Assembly (TSA) data when available [17, 21]. The tables show which species and paralogs were validated. The first number indicates the number of genes found per family (same as Supplemental file 2); the smaller characters represent EST/TSA data for each paralog. “@” indicates that a full-length or partial expression read fragment was found, “&” indicates a full-length or partial transcriptome read, “-” indicates no EST/TSA support was found. “/” separates multiple paralog gene copies (a, b, c, d) “,” indicate multiple transcript variants exist which include different exons (.1,.2), “*” indicates EST/TSA data did not include exon sequences for respective alternative transcripts (.1,.2). Dark blue/orange boxes indicate all paralogs were validated by partial or full EST/TSA hits, light blue/orange boxes indicate no reads were found to support that paralog. White boxes indicate that no EST or TSA data were available for analysis. Red boxes indicate EST and TSA data were found without sequence evidence for the gene present within the genome assembly. A) EST data. B) TSA data. (PNG 1711 kb) [file 12862_2018_1147_MOESM8_ESM.png]

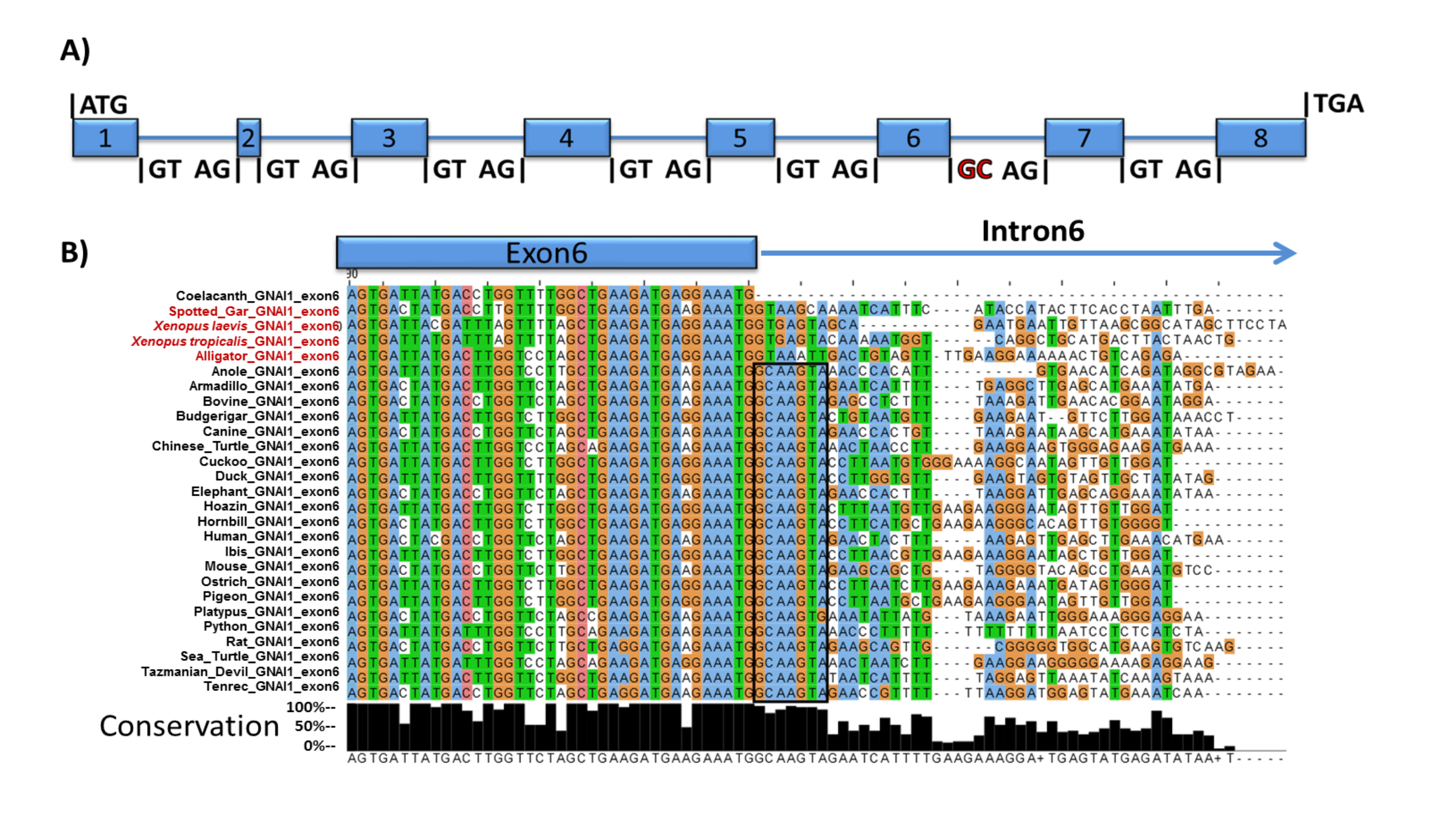

Supplement: Supplementary file 9 — Figure S7. 5′ non-canonical splice site pattern of GNAI1 intron6 in Sauropsida and Mammalia. A) Schematic representation of the primary transcript sequence of the GNAI1 gene in Sauropsida and Mammalia with the start and stop codons as well as the SS explicitly shown. Possible untranslated regions (UTRs) are not shown. The representative exons (boxes) are drawn to approximate scale with their nucleotide length while introns (lines) are not drawn to scale. B) 5’ SS of intron6 in GNAI1 of Sarcopterygii and spotted gar. The first seven nt of intron6 are highly conserved in all Mammalia and most Sauropsida (black box), while they vary in alligator, frogs and spotted gar (species marked in red). The intron sequence, and thus SS, is unknown for coelacanth. The first two nt of the boxed region constitute the SS pattern GC/GT. The figure was produced with the Jalview alignment viewer [30]. (PNG 2608 kb) [file 12862_2018_1147_MOESM9_ESM.png]

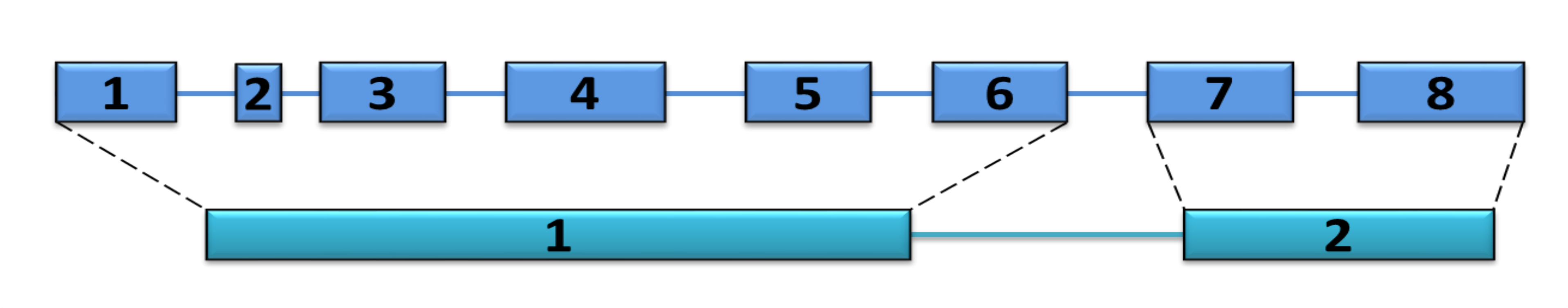

Supplement: Supplementary file 10 — Figure S2. Exon structure of GNAI and GNAZ. Most members of the Gαi family have a conserved gene structure with 8 protein-coding exons, similar exon lengths, and five conserved split codons shared across exons. The relative exon lengths of GNAI genes are represented by dark blue boxes. GNAZ only possesses two protein-coding exons (light blue). The first GNAZ exon sequence maps to exons 1–6 of GNAI, while the second GNAZ exon position maps to exons7 and 8 of GNAI. This exon-intron structure is indicative of a retrotransposition. The intron sequence may have been reinserted later into the gene to promote transcription. (PNG 152 kb) [file 12862_2018_1147_MOESM10_ESM.png]

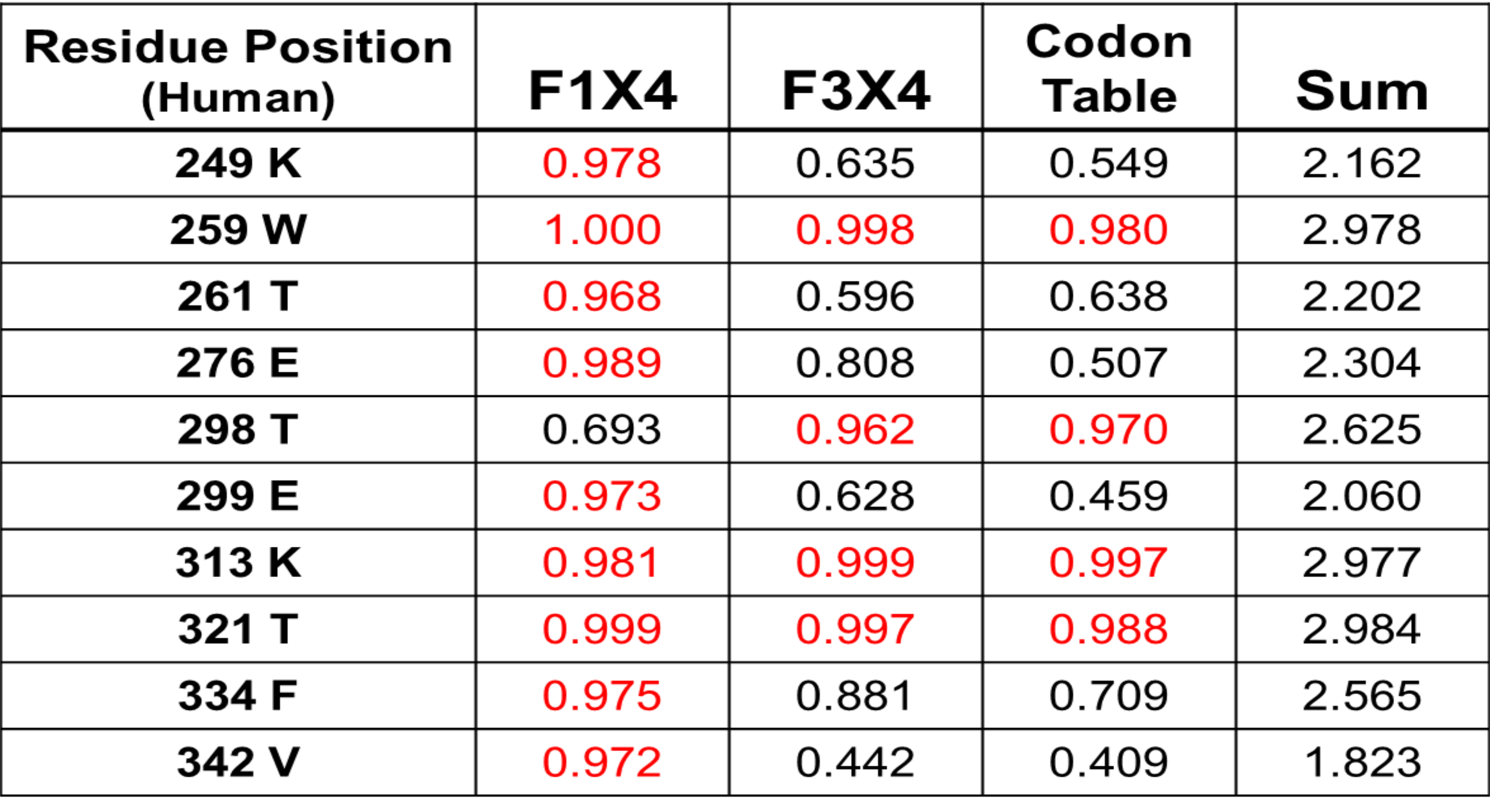

Supplement: Supplementary file 11 — Table S2. Sites under positive selection in the branch leading to GNAO.1. Data is given for those residues that have a BEB probability for being in class 2a (sites under positive selection) for branch #1 (Fig. 9) > 90% in at least one of the tested codon models (F1X4, F3X4, Codon Table). The probabilities > 90% are marked in red. The identity and numbering of the residues in respect to the full-length protein sequence in human are given in column 1. (PNG 1046 kb) [file 12862_2018_1147_MOESM11_ESM.png]

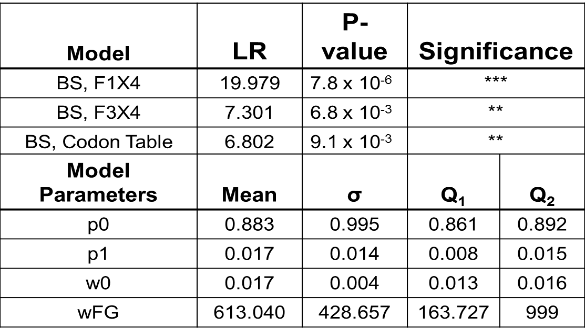

Supplement: Supplementary file 12 — Table S3. Significant results of the branch-site model indicate positive selection in the GNAO.1 #1 branch. The result of the likelihood ratio test was compared to a χ2 distribution with following significance levels * < 0.05, ** < 0.01, *** < 0.001 for each codon model tested (F1X4, F3X4, codon Table) in the #1 branch of GNAO.1 (marked in Fig. 9). All other tested branches (#2, *1, *2, and *3) were not significant. Robustness of the parameter inferences (p0, p1, w0, wFG) was accessed by bootstrapping. BS = Branch-Site, LR = Likelihood Ratio, σ = standard deviation, Q1 = First Quantile (25th percentile), Q2 = Second Quantile (75th percentile). (PNG 52 kb) [file 12862_2018_1147_MOESM12_ESM.png]

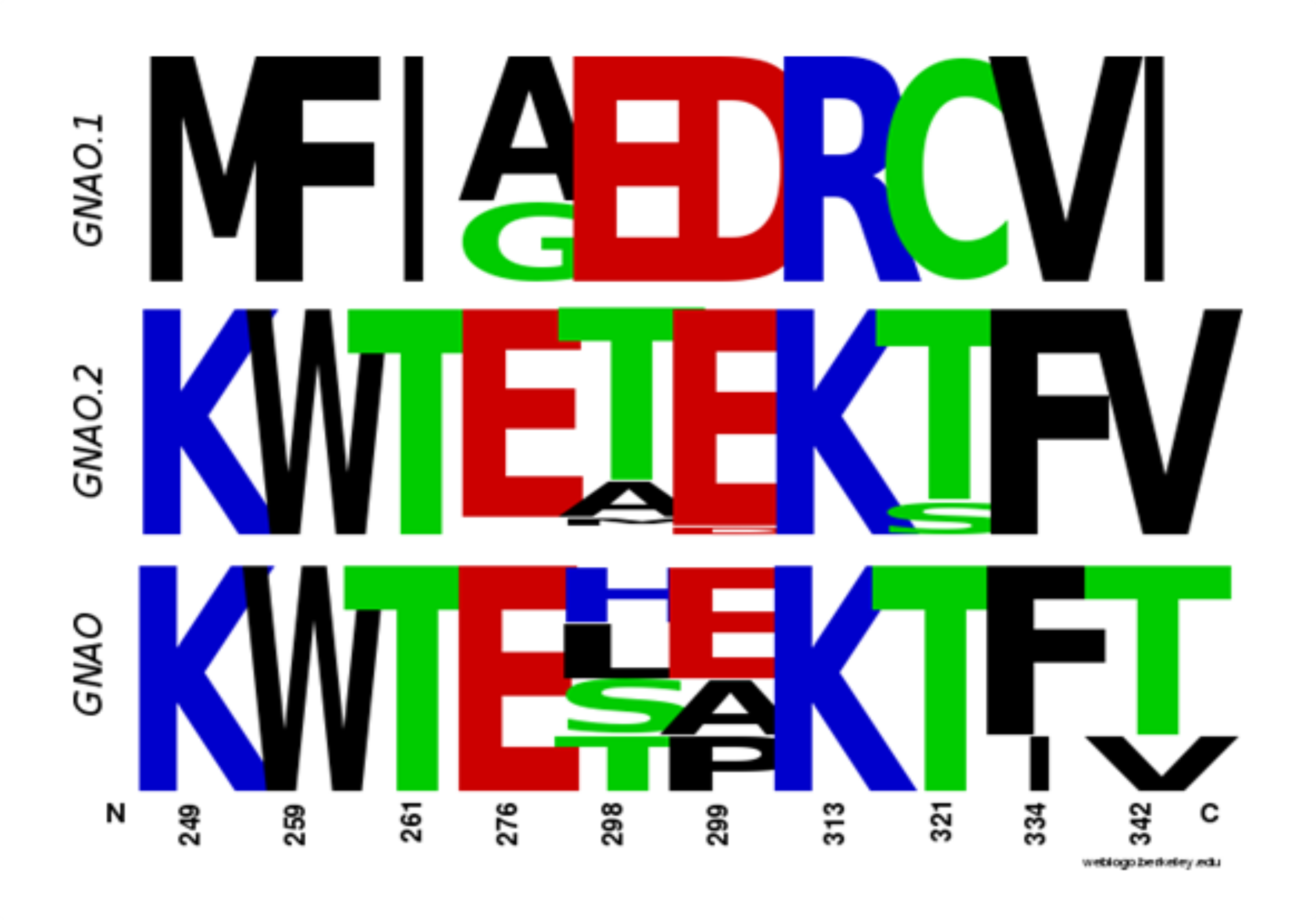

Supplement: Supplementary file 13 — Figure S3. Sequence frequency logo of GNAO residues that were positively selected on the branch leading to GNAO.1. The duplication resulted in two pairs of exons7–8 that are mutually exclusive during splicing. Alternative splicing produces two transcript variants, GNAO.1 and GNAO.2, that slightly differ in sequence. Some residues of the GNAO.1 branch were positively selected after the duplication (branch #1 of Fig. 9). The identity of homologous positions is also shown for GNAO of Hemichordata, Echinodermata and Cephalochordata (lowest track). Teleostei and lampreys were excluded when testing for positive selection and when constructing the sequence logo. The sequence logo was created with Weblogo [123]. (PNG 824 kb) [file 12862_2018_1147_MOESM13_ESM.png]

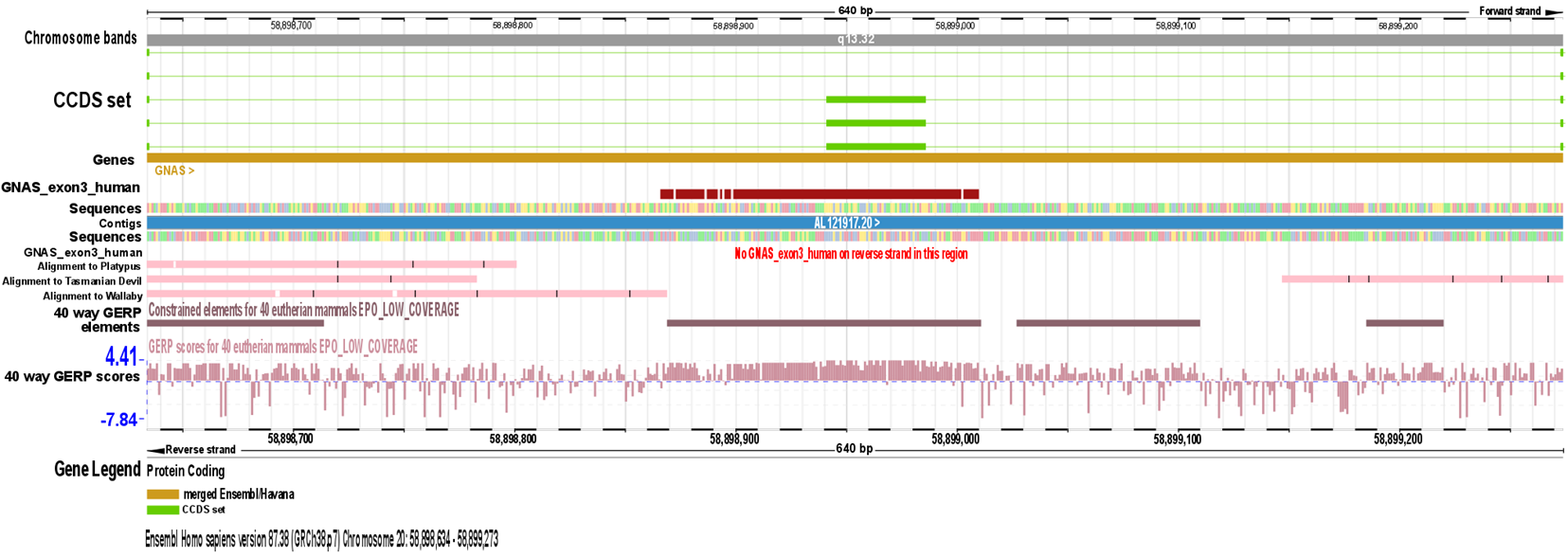

Supplement: Supplementary file 14 — Figure S4. Exon 3 of GNAS in human. Expression of exon3 is supported by CCDS data. A region ~ 75 nt upstream and 25 nt downstream of the exon boundaries shows high levels of conservation in Placentalia. The same region is not conserved in non-placental Mammalia (platypus, wallaby and Tasmanian devil) as no BLASTz hits were retrieved (pink boxes). The Figure was created with the Ensembl webserver [15]. Bp - Basepair, CCDS - consensus coding sequence, GERP - Genomic Evolutionary Rate Profiling. (PNG 249 kb) [file 12862_2018_1147_MOESM14_ESM.png]

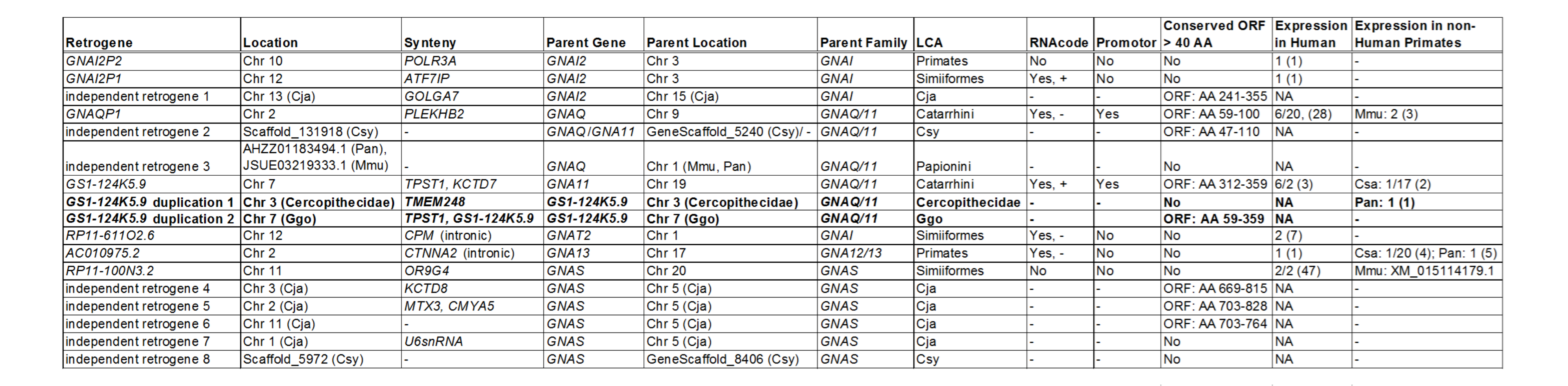

Supplement: Supplementary file 15 — Table S4. Retrogenes in Primates. The table summarizes the properties of GNA- retrogenes found in Primates. Two retrogenes (highlighted in bold) are the result of independent duplications of an existing retrogene. All other retrogenes are the result of a retrotransposition event. The retrogene name, location, location of the parent and the proximity to a promotor are given for human unless specified differently in parenthesis. The retrogene is situated next to the gene specified in the synteny column for the phylogenetic group given in the column ‘LCA’ (last common ancestor). Requiring conservation within the complete phylogenetic group, the coding potential of the respective region was evaluated with RNAcode 40] (+: methionine contained in open reading frame, ORF; −: no methionine in ORF). Conserved ORFs that are similar to the parent ORF were detected via blastn with the human parent gene as query. Expression was accessed by interrogating the Expression atlas database restricting to RPKM > 0.5 and additionally other sources for non-human Primates. Given is the number of experiments, the number of conditions (in parenthesis) and the number of tissues (last value) in the last two columns. Cja – Callithrix jacchus, Ggo – Gorilla gorilla, Csy – Tarsius syrichta, Mmu – Macaca mulatta, Pan – Pongo abelii, Csa – Chorocebus sabaeus. (PNG 1280 kb) [file 12862_2018_1147_MOESM15_ESM.png]

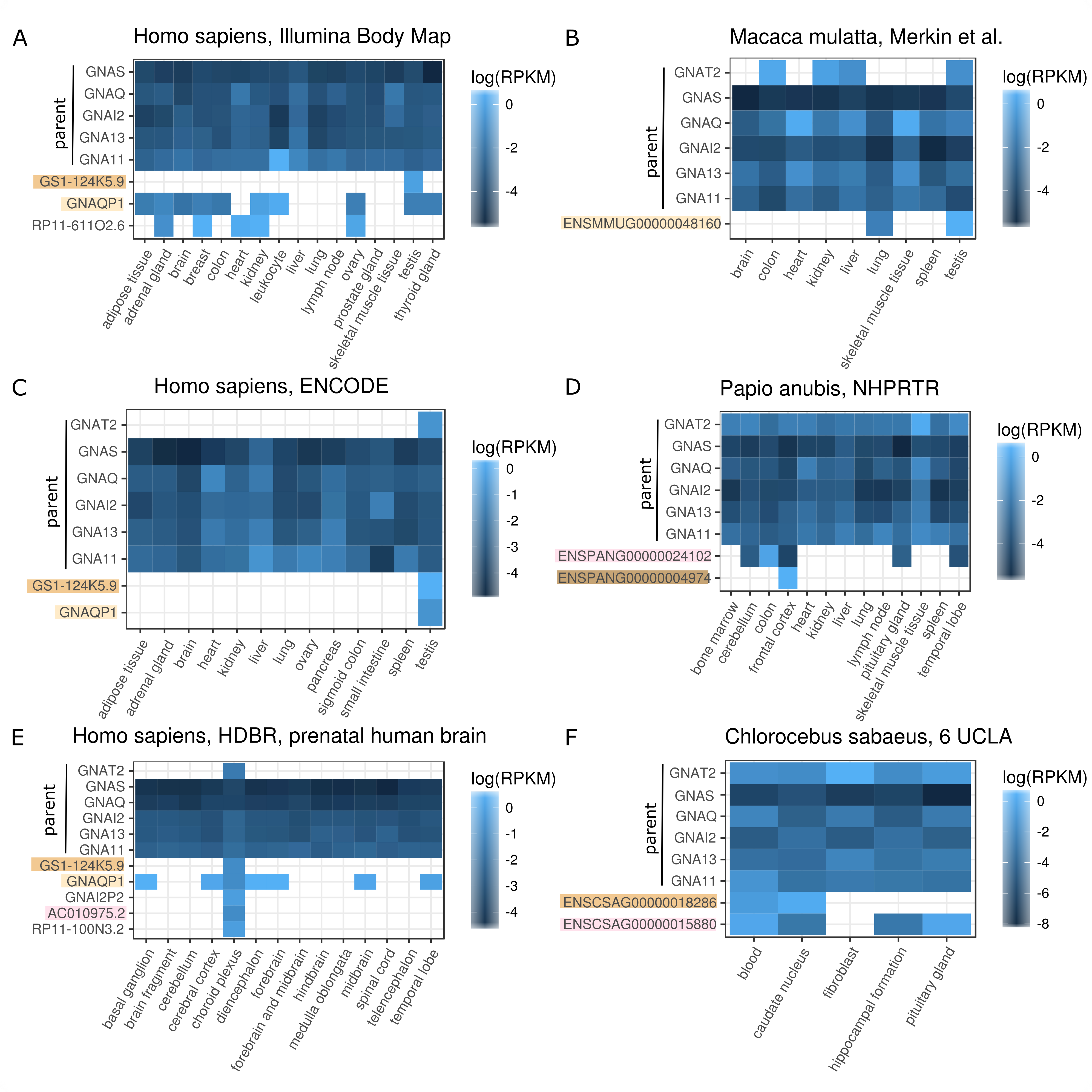

Supplement: Supplementary file 16 — Figure S5. Expression level heatmap of GNA-retrogenes and parent genes in different Primates. We depict a selection of RNA-Seq datasets which show expression of the respective GNA- retrogenes with RPKM > 0.5. The color scheme depicts orthology relationships. Note that the dark brown paralog is the results of an independent duplication of GS1-124 K5.9. A) RNA-Seq experiment of 16 human individual tissues and mixture from the Illumina Body Map (primarily Caucasian origins from both sexes, ages 19–86) [124]. B) RNA-Seq experiment of 9 rhesus macaque tissues from Merkin et al. (male, unknown age) [125]. C) RNA-Seq experiment of 13 human tissues from the ENCODE project (both sexes, 21–66 years) [126]. D) RNA-Seq of 14 tissues of olive baboon from the non-human Primates reference transcriptome resource project (female, 6 years) [127]. E) RNA-Seq experiment in 14 human brain tissues from the Human Developmental Biology Resource (both sexes, 10 weeks post conception) [128], F) RNA-Seq experiment of 5 vervet-AGM tissues (male, 3 years). (PNG 1640 kb) [file 12862_2018_1147_MOESM16_ESM.png]

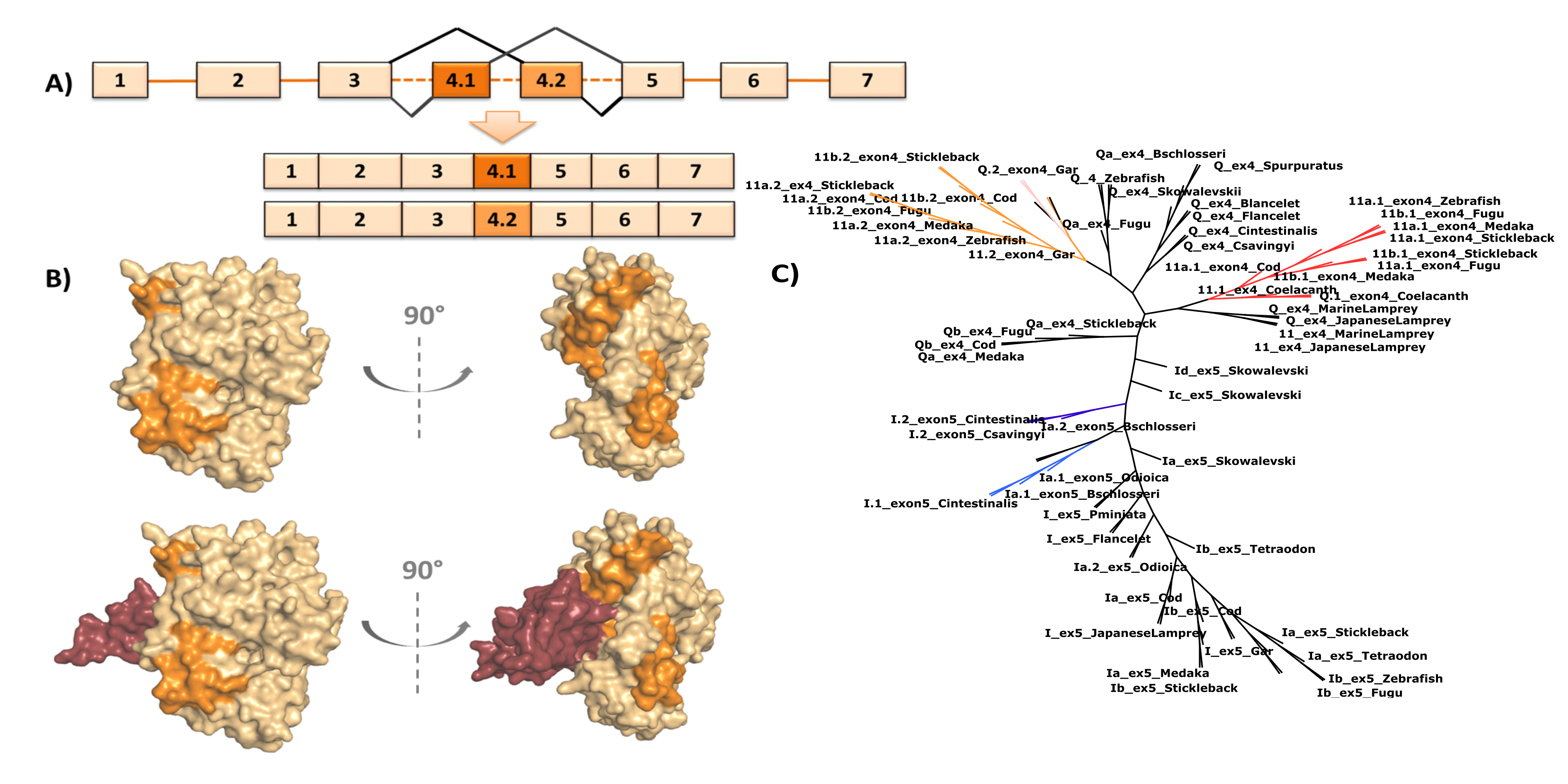

Supplement: Supplementary file 17 — Figure S6. Local exon duplications of GNAQ, GNA11, and preGNAI. A) Alternative splicing of two mutually exclusive exon4 of GNAQ and GNA11 results in two different RNA transcripts represented. Box lengths correlate with average curated exon lengths (intron line lengths do not correspond to intron lengths). B) Tertiary crystal structure of Mammalia Gαq (taupe) with exon4 (orange) borders mapped with RGS protein interaction removed (top) and with RGS present (bottom - ruby) (PDBID 5D09 [54]). Alternatively, spliced exon4 provides sequence diversity for critical protein-protein interfaces such as the RGS protein (purple). C) ML trees of nucleotide sequences from exon4 of GNAQ/GNA11and exon5 of GNAI across basal Chordata. (PNG 1933 kb) [file 12862_2018_1147_MOESM17_ESM.png]

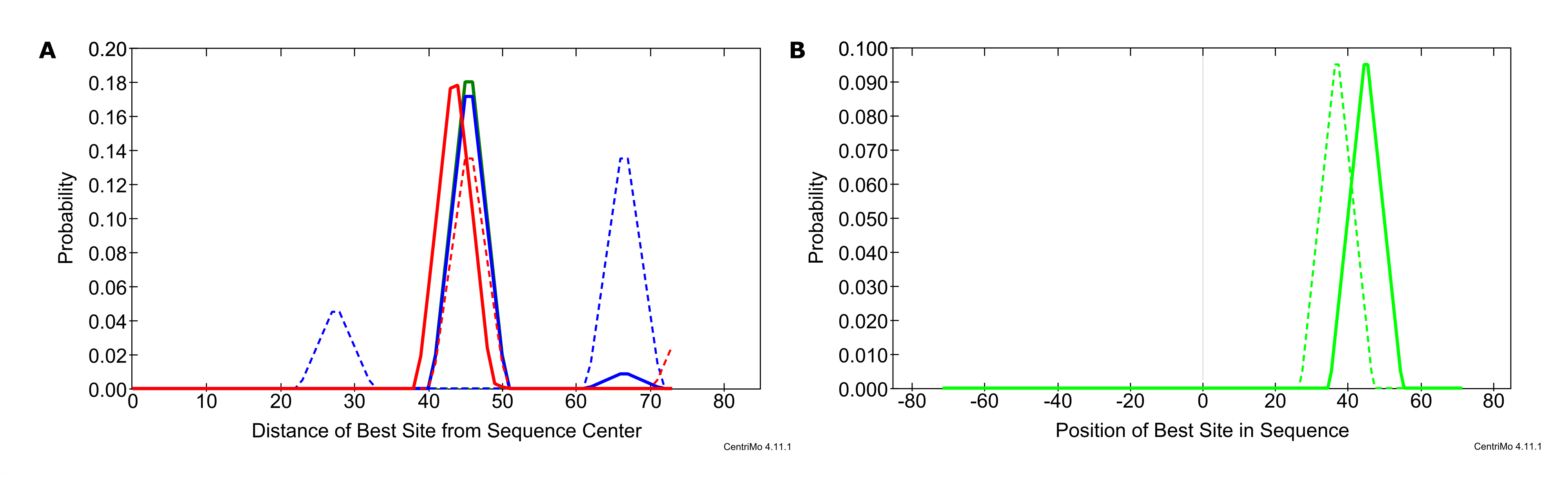

Supplement: Supplementary file 18 — Figure S8. DNA- and RNA-binding protein motifs overlapping with the 5′ non-canonical splice site of intron6 in GNAI1. A) Local enrichment of known DNA-binding protein (DBP) motifs in comparison to a uniform motif distribution are shown for Sarcopterygii with ‘GC’ SS (positive set) versus lobe-finned fish and spotted gar with ‘GT’ splice site (SS) (control, adjusted p-value < 0.05). The shown motifs are either present in all species of the positive set and in none of the controls (PRDM1_full, FXR1) or follow this rule with at most one exception. Mafk_secondary UP0004_2 (red), NFIX_full_3 (dark blue), PRDM1_full (green), STAT2:STAT1 (pink, behind green). B) Local enrichment of known RNA-binding protein (RBP) motifs in comparison to a uniform motif distribution. FXR1 (lime green). The SS is located at position 45 along the x-axis. Sequence positions < 45 correspond to exon6, while positions > 45 correspond to intron6. The y-axis indicates the probability of a DBP/RBP motif present centrally at the indicated position for the positive set (solid line) and the control set (dotted line). None of the motifs occurs surprisingly more often at a specific position in the positive set than in the control set (Fisher’s exact test, adjusted p-value < 0.05). The Figure was created with Centrimo [36]. (PNG 287 kb) [file 12862_2018_1147_MOESM18_ESM.png]

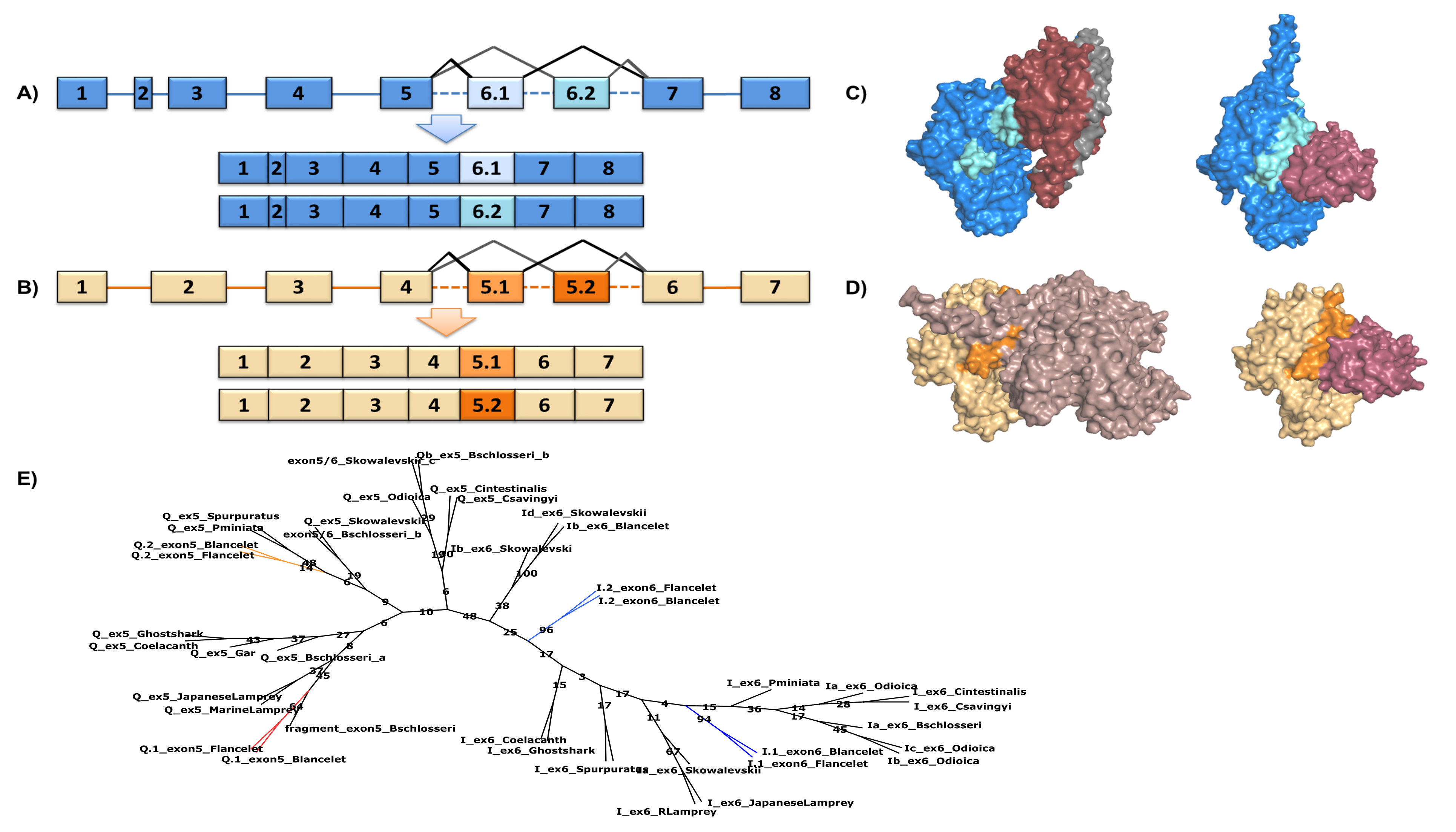

Supplement: Supplementary file 19 — Figure S9. Implications of alternative exon usage on tertiary structure in Cephalochordata preGαi and preGαq. A) Mutually exclusive inclusion of Cephalochordata exon6.1 and 6.2 in preGNAI (blue) yields two different transcripts during alternative splicing. Representative box lengths correlate with the average curated exon lengths (intron lines do not). B) Mutually exclusive inclusion of Cephalochordata exon5.1 and 5.2 in preGNAQ (beige) also yields two different transcripts during alternative splicing. C) Splice variant exon borders mapped onto two Gαi crystal structures (PDB IDs 1GP2 [56] and 1AGR [57], respectively). The sequence encoded by exon6 (light blue) influences the interface between the Gβγ subunits of the heterotrimer (crimson/grey - left) and downstream effector protein partners such as the RGS protein (purple – right). D) Splice variant exon borders mapped onto two Gαq crystal structures. The sequence encoded by exon5 (orange) influences the protein interfaces between effector proteins such as PLC (lavender – left) and RGS (purple - right) (PDB IDs 4QJ3 [53] and 5DO9 [54]). E) ML tree of (pre)GNAI/GNAQ exons indicates both duplications were independent. (PNG 1799 kb) [file 12862_2018_1147_MOESM19_ESM.png]

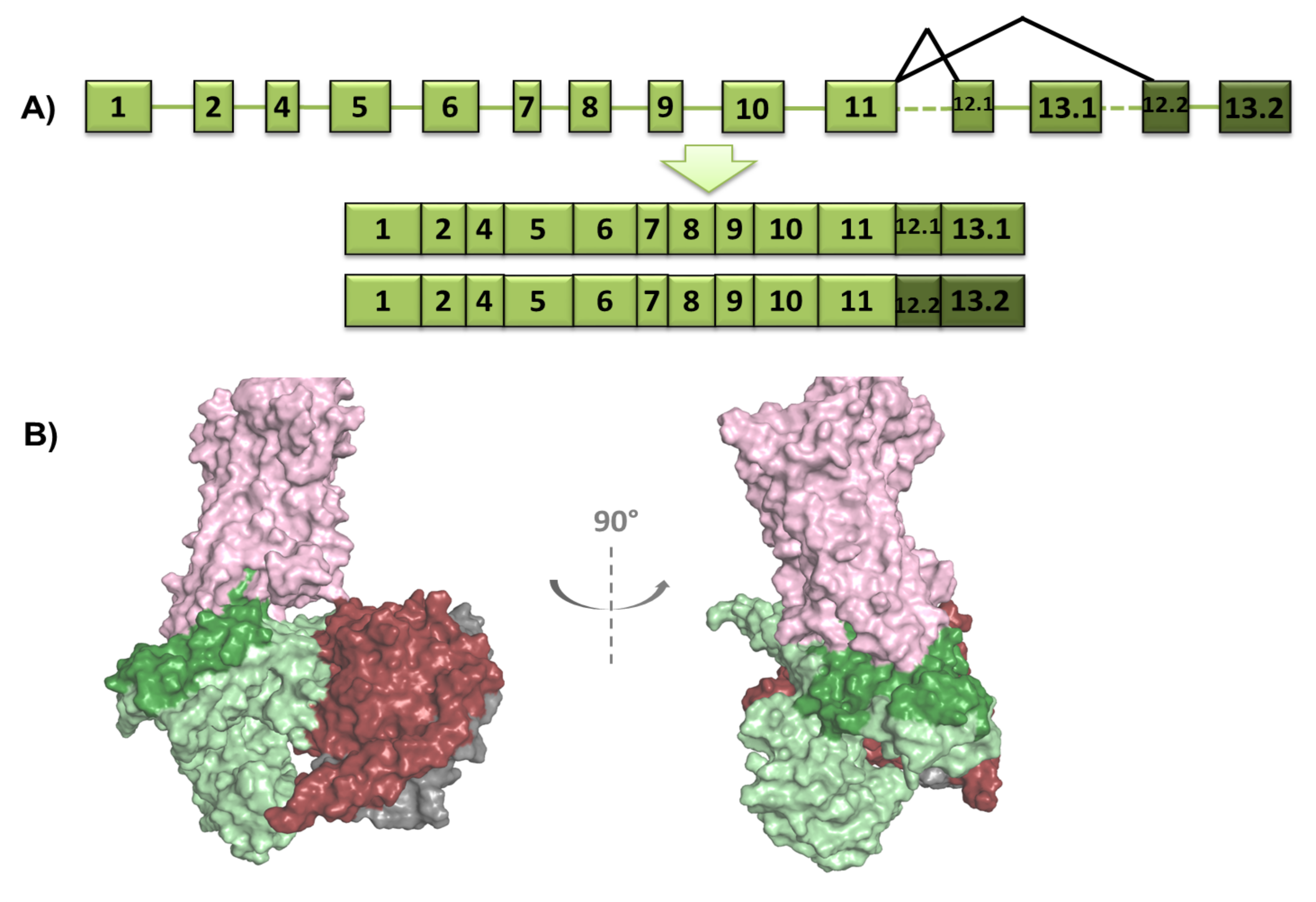

Supplement: Supplementary file 20 — Figure S10. Implications of alternative exon usage on tertiary structure in Cephalochordata preGαs. A) Alternative splicing of Cephalochordata exon12 and 13 in preGNAS (green) yields two different mutually exclusive transcripts. B) Splice variant exon borders (dark green) mapped onto a Gαs structural model bound to the G protein βγ subunits (crimson/grey) and a GPCR (pink) respectively and rotated 90°. (PNG 2085 kb) [file 12862_2018_1147_MOESM20_ESM.png]

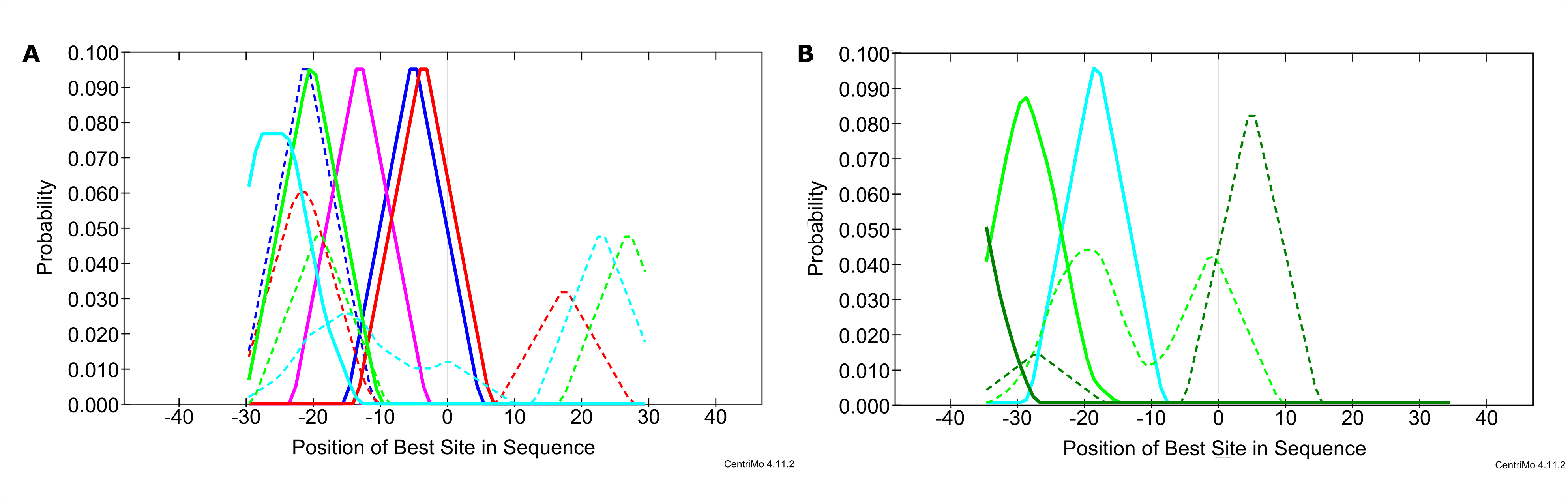

Supplement: Supplementary file 21 — Figure S11. DNA- and RNA-binding protein motifs overlapping with the 3` canonical and non-canonical splice sites of intron 3 in GNAS. All included motifs are predicted to occur in the positive set (for six Placentalia), but not at the same position in the control set (eight non-Placentalia Sarcopterygii). Note, that some motifs occur in the control set, but at a different position than in the positive set, e.g. Gata4. The shown motifs overlap with the conserved intronic region upstream of exon 4. A) Local enrichment of known DNA-binding protein motifs (DBP) in comparison to a uniform distribution of motifs (E-value < 1, adjusted p-value < 0.05). Gata4 (blue), Mybl1_secondary (pink), GATA3_full (red), Sox4_secondary (green), FOXP1 (turquoise). B) Local enrichment of known RNA-binding protein motifs in comparison to a uniform distribution of motifs (E-value < 1, adjusted p-value < 0.05). PCBP1 (light blue), U2AF2 (light green), RBM47 (dark green). The non-canonical splice site is located at position − 7. Sequence positions < − 7 belong to intron 2 while positions > − 7 belong to exon 4. The y-axis indicates the probability of a DBP/RBP binding centrally at the indicated position for the positive set (solid lines) and for the control (dotted lines). None of the motifs occurs more often at a specific position in the positive set than in the control set (Fisher’s exact test, adjusted p-value < 0.05). The Figure was created with Centrimo [38]. (PNG 410 kb) [file 12862_2018_1147_MOESM21_ESM.png]

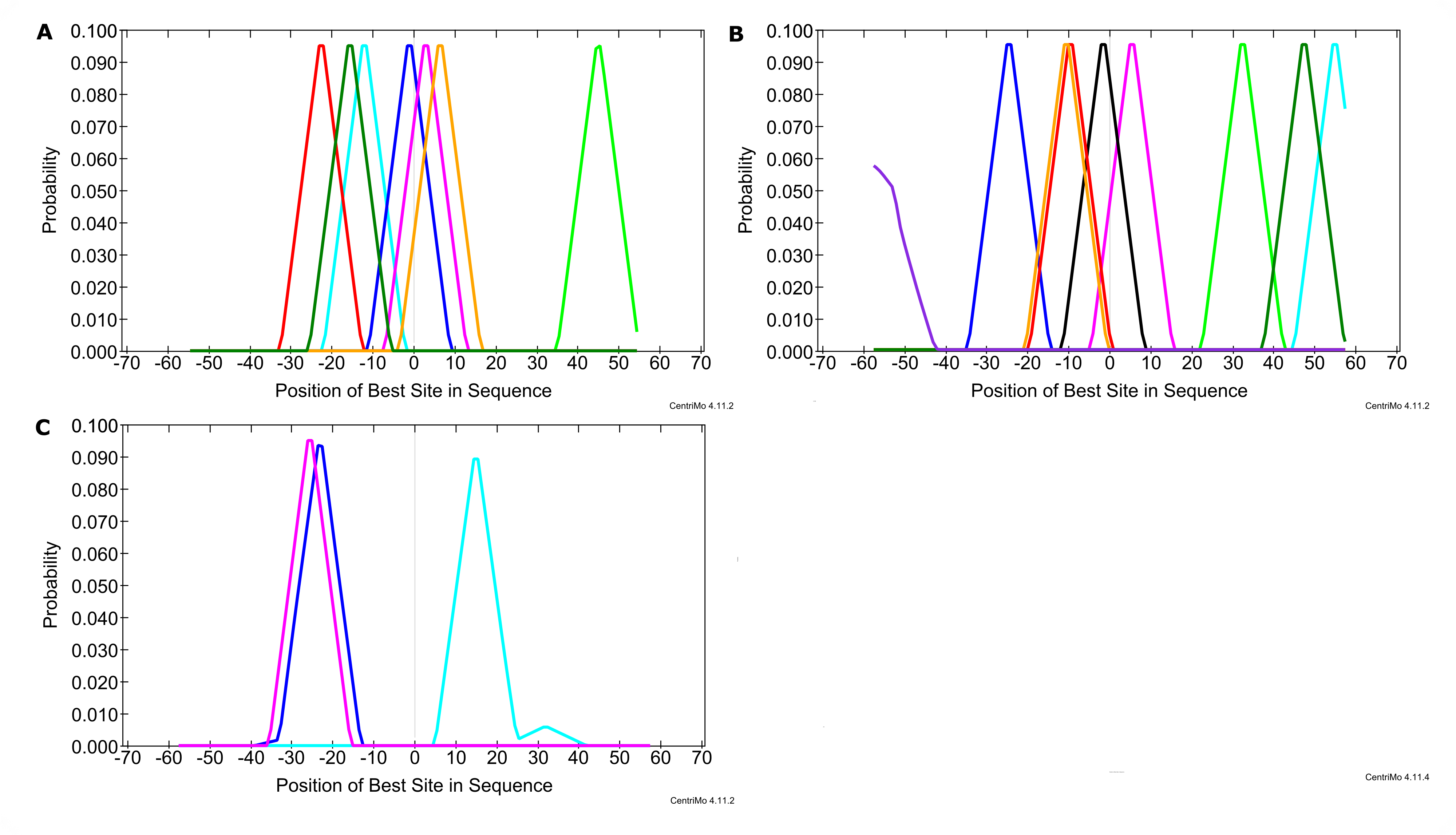

Supplement: Supplementary file 22 — Figure S12. DNA- and RNA-binding protein motifs overlapping with the extended conserved region around exon 3 in GNAS of 33 Placentalia. Exon 3 is located at positions 0–46 on the x-axis. A) Local enrichment of known DNA-binding protein (DBP) motifs in comparison to a uniform motif distribution. 30 motifs are enriched in the reported region with a E-value < 0.0001 in all investigated Placentalia; only a subset of these is shown for clarity: Gfi1 (light blue), Hltf (dark blue), EGR1 (pink), MZF1_5–13 (light green), En1 (red), E2F4 (orange), Hoxc9 (dark green). B) Local enrichment of known RNA-binding protein (RBP) motifs in comparison to a uniform motif distribution. Nine motifs are enriched in the reported region with an E-value < 0.0001 in all investigated Placentalia. TARDBP (light blue), DAZAP1 (dark blue), PPRC1 (pink), SRSF9 (light green), SRSF10 (red), CNOT4 (orange), PCBP1 (dark green), KHDRBS1 (black), RBM38 (purple). Note that the SRSF9 binding site is located within the exon and does not overlap with either splice site. C) Local enrichment of RBP sites predicted by Pollard et al. [112]. The respective motifs do not occur in all investigated Placentalia as indicated by a lower probability. SRSF2 (dark blue), SRSF1 (light blue), HNRNPA1 (pink). The 3′ ‘AG’ SS is located at position 0 along the x-axis. The y-axis indicates the probability of a DBP/RBP motif being located centrally at this position. The Figure was created with Centrimo [38]. (PNG 598 kb) [file 12862_2018_1147_MOESM22_ESM.png]
